# Simultaneous detection of small molecule thiols with a simple ^19^F NMR platform†

**DOI:** 10.1039/d0sc04664g

**Published:** 2020-11-13

**Authors:** Zhaofei Chai, Qiong Wu, Kai Cheng, Xiaoli Liu, Ling Jiang, Maili Liu, Conggang Li

**Affiliations:** Key Laboratory of Magnetic Resonance in Biological Systems, State Key Laboratory of Magnetic Resonance and Atomic and Molecular Physics, National Center for Magnetic Resonance in Wuhan, Wuhan National Laboratory for Optoelectronics, Wuhan Institute of Physics and Mathematics, Innovation Academy for Precision Measurement Science and Technology, Chinese Academy of Sciences Wuhan 430071 China conggangli@apm.ac.cn; Graduate University of Chinese Academy of Sciences Beijing 100049 China

## Abstract

Thiols play critical roles in regulating biological functions and have wide applications in pharmaceutical and biomedical industries. However, we still lack a general approach for the simultaneous detection of various thiols, especially in complex systems. Herein, we establish a ^19^F NMR platform where thiols are selectively fused into a novelly designed fluorinated receptor that has two sets of environmentally different ^19^F atoms with fast kinetics (*k*_2_ = 0.73 mM^−1^ min^−1^), allowing us to generate unique two-dimensional codes for about 20 thiols. We demonstrate the feasibility of the approach by reliably quantifying thiol drug content in tablets, discriminating thiols in living cells, and for the first time monitoring the thiol related metabolism pathway at the atomic level. Moreover, the method can be easily extended to detect the activity of thiol related enzymes such as γ-glutamyl transpeptidase. We envision that the versatile platform will be a useful tool for detecting thiols and elucidating thiol-related processes in complex systems.

## Abstract 2

A ^19^F NMR platform, capable of discriminating various small molecule thiols, was designed for in-cell thiol differentiation and monitoring, and further detection of the γ-GT activity, demonstrating the wide applications in thiol-related processes.

## Graphical abstract

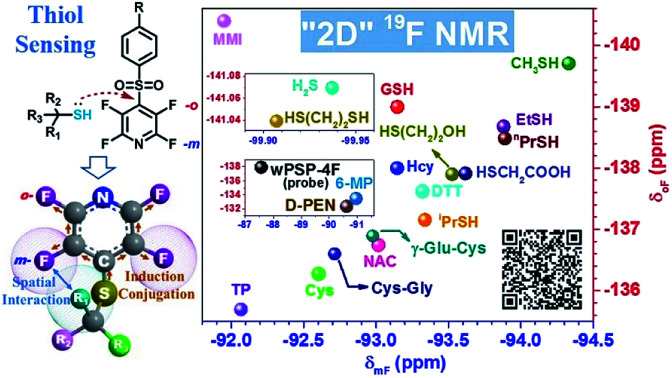

## Introduction

Small molecule thiols are of great interest to the scientific community owing to their vital roles in many physiological processes and pharmaceutical applications. For example, biothiols of cysteine (Cys), homocysteine (Hcy), glutathione (GSH) and hydrogen sulfide (H_2_S) are found in abundance and involved in many cellular regulations, and their abnormal levels are directly correlated with many different diseases.^1–3^ Meanwhile, thiol drugs (*e.g.* penicillamine, tiopronin, and thiamazole) are present with endogenous thiols to display different curative effects in the clinical field.^4–6^ Thus, real-time identification and quantification of these thiols would be of considerable significance to provide insights into related pathophysiological changes and pharmacokinetic information. Consequently, various analytical methods have been reported to sense and image these species in living cells and organisms, especially simultaneous detection of two or more species, which could provide more information on their close relationship under specific metabolism and joint action in disease diagnosis.^7–9^ So far, the noninvasive and selective detection of thiols mainly focuses on the fluorescence technology, involving efficient probes with multiple reaction sites or cleavage paths to different functional groups of the analytes. But the intrinsic property of this strategy makes it difficult for probes to discriminate those containing only one reactive sulfhydryl unit or ones with the same reaction features. To reflect the structural character and concentration of the analytes, the ^19^F NMR technique, an alternative approach, has recently emerged to identify structurally similar molecules in complex mixtures, benefitting from the similar gyromagnetic ratio of ^19^F to ^1^H, 100% natural abundance of ^19^F, scarcity of naturally occurring background signals, and a broad chemical shift range.^10,11^ Though the transformation of similar structural information of analytes into resolvable ^19^F NMR shifts is intriguing, the probe design is still far from being fully explored. Only a few fluorinated compounds have been successfully used to discriminate biological organic compounds in simulated or simple biological samples.^12–17^ So the systematic design of probes for multiple thiol discrimination is valuable for biological research and ^19^F NMR probe engineering.

In this study, based on the nucleophilic substitution of –SH towards fluorinated sulfoxides, we introduced a simple ^19^F NMR platform that enabled the recognition of around 20 thiols with high sensitivity and rapid reaction kinetics under physiological conditions. The new method successfully achieved endogenous thiol quantification and the real-time monitoring of their conversion in living HeLa cells. Importantly, we observed a critical intermediate during GSH (or GSH–probe adduct) metabolism in living cells, which would help improve the accuracy of further Cys and GSH measurements. We also extend the proposed strategy to detect thiol-related enzymes, such as γ-glutamyl transpeptidase (γ-GT).

## Results and discussion

### Design of the ^19^F probe with rapid response, high sensitivity, and reliable quantification

**Fig. 1:**
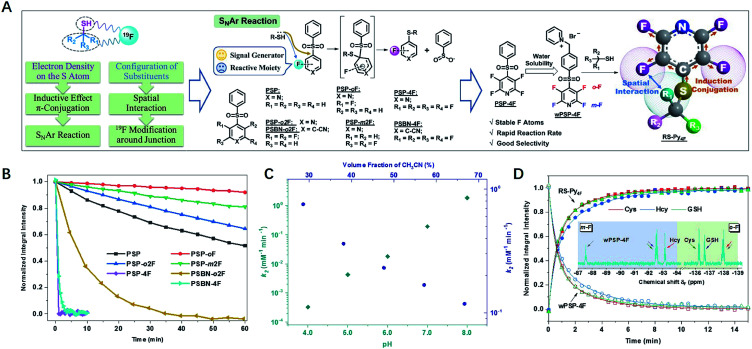
(A) The illustration of the principles of probe design and related probes. (B) Comparison of reaction rates between sulfones (1 mM) and Cys (10 mM) in PB (100 mM, pH = 7.40, 10% D_2_O, 30% AN, rt). (C) The second-order rate constants (*k*_2_) between PSP-4F and Cys under different pH values and volume fractions of CH_3_CN. (D) The time-dependent integral intensity of wPSP-4F (1.0 mM) and products (RS–Py_4F_) upon addition of Cys, GSH and Hcy (1.5 mM) in PB (100 mM, pH = 7.40, 10% D_2_O, rt). Inset shows the ^19^F NMR spectra of the mixture of wPSP-4F and the biothiols.

Our ^19^F NMR approach needed ^19^F probes with high sensitivity to the local electronic microenvironment induced by thiols (Fig. 1A).^18^ In order to transmit the electron density on the sulfur atom through both inductive effect and conjugation more efficiently, we chose nucleophilic aromatic substitution (S_N_Ar) that fluorinated aromatic sulfones and thiols formed stable thioethers.^19–21,43–47^Another characteristic of substitution arrangement around –SH was that it could be perceived by adjacent ^19^F atoms *via* spatial proximity. To develop a practical probe with fast response and good selectivity, we synthesized an array of F-substituted sulfones through a one-step process (ESI†), and evaluated their practicability with three typical biothiols of Cys, Hcy, and GSH. The results indicated that the modification of electron-withdrawing units (F atoms, pyridyl, and benzonitrile) could significantly increase the reactivity of sulfones (Fig. 1B, S1–S8†). Both PSP-4F and PSBN-4F outstood among these candidates because of their short reaction times (<3 min). Owing to the strongest electron-deficient ability, PSBN-4F displayed a very complicated ^19^F NMR spectrum upon the addition of Cys due to the activated F–C bonds and the special S → N rearrangement,^22–25^ while PSP-4F formed a stable product and exhibited two explicit peaks under the same conditions, which was much more straightforward for further analysis. Besides suitable reactivity, PSP-4F possessing two types of environmentally different ^19^F atoms also showed superior recognition ability towards thiols (Fig. S9†). Two factors contributed to this recognition ability: the *meta*-fluorine (*m*-F) could sense the change of electron density (Cys *vs.* Hcy) and meanwhile kept the thiol signals away from the probe itself (Δ*δ* > 4 ppm), and the *ortho*-fluorine (*o*-F) was able to precisely reflect the structural variation around –SH owing to different interactions with substituents (Cys *vs.* GSH). Further investigation of PSP-4F and Cys showed that high pH values and water fraction were also favorable to this reaction, possibly due to the enhanced nucleophilicity of –SH and the stabilization of the intermediate, respectively (Fig. 1C, S10 and S11†).^25^

In order to be more compatible with biological samples, PSP-4F was converted into a water-soluble pyridinium salt (wPSP-4F), which likewise achieved satisfactory discrimination with fast reaction kinetics under physiological conditions (Fig. 1D, S12 and S13†). wPSP-4F also manifested good selectivity towards –SH (Fig. S14†), and common inorganic ions and amino acids except for Cys did not induce any observable changes. Only H_2_S elicited a set of sharply distinguishable peaks compared with three biothiols (Δ*δ*_*m*F_ > 7 and Δ*δ*_*o*F_ > 3 ppm). Moreover, two distinct peaks (Δ*δ* > 50 ppm) were beneficial to further multi-thiol determination through selective peaks (*m*-F or *o*-F) with better resolutions. In addition, the integral intensities of wPSP-4F and corresponding thioether (RS–Py_4F_) were linearly proportional to the thiol concentration (*R*^2^ > 99.7%) in the 0.1–10 mM range (GSH for instance), in favor of quantitative detection (Fig. S15†). Compared with reported ^19^F NMR probes for multi-component detection, our probe reacted with thiols in a stoichiometric proportion of 1 : 1 and formed stable thioester without any exchange with analogs, which improved the detection limit (<5 μM) under the same conditions (Fig. S16†) and widened its potential application in complex environments.^13–16^

As an illustration of reliable quantitative detection, the content of thiols in commercial *N*-acetylcysteine (NAC) effervescent tablets was determined (Table 1, Fig. S17 and S18†).^26–28^ By simply mixing the probe and samples, the NMR approach gave similar accuracy to the standard HPLC method,^29^ and NMR could determine the NAC at a lower concentration (0.5 mg mL^−1^*vs.* 0.01 mg mL^−1^) of NAC, suggesting wPSP-4F to be a promising and practical tool for thiol quantification *in vitro*.

**Table 1:** Determination of NAC in drugs

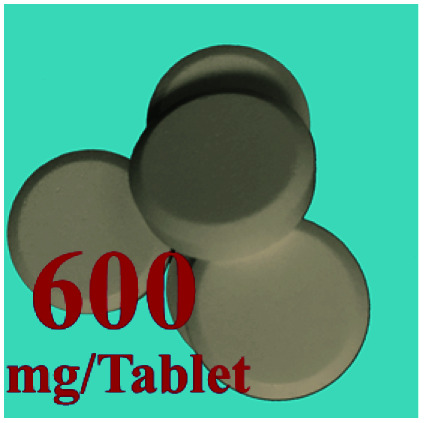	Method	Found (mg)	RSD (‰)	Recovery (%)
	HPLC	568	8.3	—
	^19^F NMR	575	7.9	1.01

### Distinct discriminatory ability of the ^19^F probe towards small molecule thiols

**Fig. 2:**
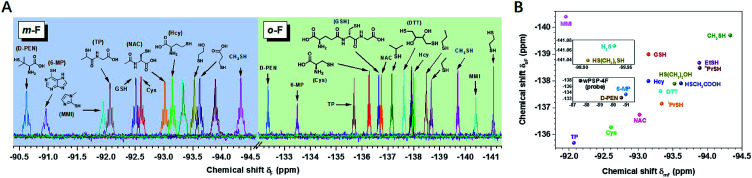
(A) ^19^F NMR spectra of wPSP-4F (100 μM) upon addition of thiols (10 mM) in PB (100 mM, pH = 7.40, 10% D_2_O, rt). (B) 2D code of different thiols. Note that EtSH, ^*n*^PrSH, ^i^PrSH, and (CH_2_SH)_2_ were added to the system with 2 vol% DMSO. The chemical shifts were calibrated by the internal reference of TFA.

After the initial test of the probe with typical biothiols, we assessed its discriminatory ability with a variety of other biothiols, thiol drugs, and pollutants that may coexist or have closely related structures. As shown in Fig. 2A, most of the thiols showed well-resolved *m*- or *o*-F peaks, except a few similar structures that had overlapped ones. However, the ones with overlapped *m*-F peaks had separated *o*-F peaks, and *vice versa*. For example, ^*n*^PrSH and EtSH displayed almost the same *δ*_*m*F_ because the electronic densities of –SH in them were similar, but they had different *δ*_*o*F_ because ^*n*^PrSH had slightly larger steric hindrance than EtSH, resulting in a downfield chemical shift of *δ*_*o*F_ (Δ*δ*_*o*F_ > 0.2 ppm). We found that the increased spatial interactions from the primary thiols to the secondary and tertiary ones led to a similar trend of chemical shifts in each region (*e.g. δ*_d-PEN_ > *δ*_TP_ > *δ*_Cys_; *δ*_i-PrSH_ > *δ*_*n*-PrSH_), but had a more profound influence on the *o*-F. In contrast, 3-mercaptopropionic acid and 2-mercaptoethanol exhibited a similar interaction with *o*-F after the reaction, but the different inductive effects of hydroxyl and carboxyl groups resulted in the change of electronic densities on the S atom and induced distinguishable δ_*m*F_s. The aromatic thiols also had characteristic fingerprints, of which 6-mercaptopurine (6-MP) with larger steric hindrance showed downfield chemical shifts in each region compared with those of methimazole (MMI). We also noticed that thiols with larger steric hindrance (d-penicillamine, d-PEN) and decreased electron density (6-MP and MMI) required more time or higher temperature to equilibrium because of their slower reaction rates (Fig. S19 and S20†). The good capacity of wPSP-4F for multi-thiol discrimination could be presented by a two-dimensional plot (2D code) with the chemical shifts of *m*-F and *o*-F as the axes, which clearly showed no overlapped signals of the tested thiols (Fig. 2B). Certainly, wPSP-4F could discriminate more small lipophilic thiols in conjunction with organic solvents to improve the solubility of thiols and corresponding thioesters (more suitable for the PSP-4F system) (Fig. S21 and S22†).

### Real-time monitoring of thiols in HeLa cells

To further test the suitability of wPSP-4F for differentiating small molecule thiols in biological systems, we tried to measure biothiols in HeLa cells. Before the measurement, the influence of free cysteine residues in proteins was assessed with cell lysates (6 mg mL^−1^) after dialysis with a 3k cut-off membrane to remove small molecule thiols (Fig. S23†). The results indicated 15.3% consumption of the probe (0.5 mM) without any ^19^F resonances. This observation was perhaps due to both the low abundance of –SH containing proteins and the line broadening effect of macromolecules, while the ^19^F spectrum of remaining dialysate with only small molecules exhibited the ^19^F chemical shift of GSH (29.4%) and Cys (3.0%), consistent with the reported ratio range.^30^ Other thiols accounted for 0.07% of the probe without any characteristic peaks because of low content. Therefore, the ^19^F NMR approach could potentially detect intracellular Cys and GSH, which had not been realized using NMR techniques before.^31^

**Fig. 3:**
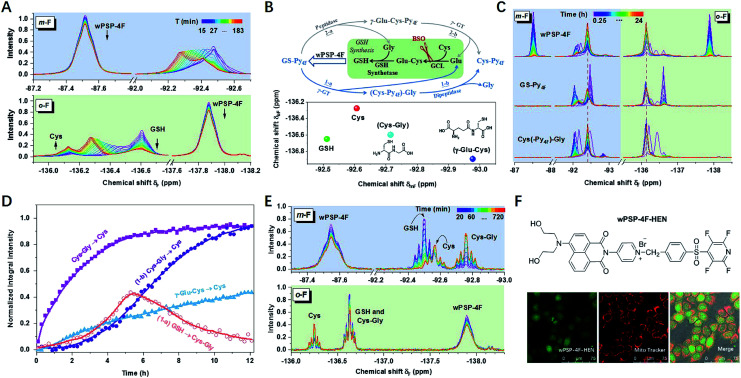
(A) Time-dependent ^19^F NMR spectra of the probe (5.0 mM) toward thiols in HeLa cells (*ca.* 4 × 10^7^, in 400 μL cell-culture medium, 10% D_2_O, rt) within 3 h. (B) Scheme of the two possible degradation pathways of GS–Py_4F_ (top) and 2D code of involved intermediates (bottom). (C) Time-dependent ^19^F NMR spectra of wPSP-4F (2.0 mM) toward thiols in HeLa cells (*ca.* 4 × 10^7^, in 400 μL cell-culture medium, 10% D_2_O, rt) within 24 h and recovery with exogenous adducts (GS–Py_4F_ and Cys(–Py_4F_)–Gly, 500 μM) under the same conditions. (D) Comparison of degradation rates of GS–Py_4F_, Cys(–Py_4F_)–Gly and γ-Glu–Cys(–Py_4F_) in the presence of fresh cell lysates at room temperature. The two-step process (1-a and 1-b) of GS–Py_4F_ degradation was calculated individually. (E) Time-dependent ^19^F NMR spectra of the probe (500 μM) with addition of HeLa cell lysates (6 mg mL^−1^) in PB (100 mM, pH = 7.40, 10% D_2_O). (F) Chemical structure of wPSP-4F–HEN (top) and corresponding confocal microscopy images of HeLa cells co-stained with MitoTracker (bottom).

In living HeLa cells, the integral intensity of the probe leveled off after 2 h at high concentrations (5.0 mM) (Fig. 3A and S24†). As expected, Cys (−136.15 ppm) and GSH (−136.60 ppm) were obviously observed in the initial stage. However, GSH underwent a rapid decline, accompanied by the increase of an unknown thiol (UT) and Cys, which was rarely addressed in any thiol-sensing system in living cells. Moreover, the long-term tracking of this process showed almost full conversion of thiols into Cys under a lower concentration (2.0 mM) with high cell viability (Fig. 3C and S25†). After ruling out the degradation of thioesters (Fig. S26†), UT was supposed to be the intermediate product during GSH (or GS–Py_4F_) metabolism. In addition, only one set of invariable peaks of Cys was detected after the cells were incubated with buthionine sulfoximine (BSO) to inhibit the activity of γ-glutamylcysteine ligase (GCL) and then GSH regeneration,^11,32^ which further confirmed our hypothesis (Fig. S27†).

Theoretically, GS–Py_4F_ had two possible degradation pathways, emerging from cleavage of the γ-glutamyl bond (γ-GT) and peptide bond (peptidase) in different orders (Fig. 3B).^33–36^ Fortunately, wPSP-4F could well distinguish the possible intermediates of γ-Glu–Cys and Cys–Gly (Fig. 3B and S28†). To reduce the line-broadening and the influence of the complex intracellular environment on NMR spectra in cells, we used diluted cell lysates to monitor the degradation of exogenous adducts (Fig. 3D and S29†). With the catalysis of endogenous enzymes, the conversion of (Cys–Py_4F_)–Gly was much more efficient than that of γ-Glu–Cys–Py_4F_, and only signals of (Cys–Py_4F_)–Gly were detected during the degradation of GS–Py_4_, in accordance with the endogenous GSH degradation in cell lysates after being treated with wPSP-4F (Fig. 3E). This process also could be stuck in the first stage of (Cys–Py_4F_)–Gly if γ-GT was added into GS–Py_4_ in dilute solution (Fig. S30†).^37^ In the further experiment, the variation trend was recovered in living cells with these adducts, though the time courses were different owing to different catalytic rates and cell permeability (Fig. 3C). The above results implied that the irreversible GSH adduct quickly decomposed to (Cys–Py_4F_)–Gly (UT) (*t*_1/2_ ≈ 76 min under our conditions) under the catalysis of γ-GT and then Cys–Py_4F_ with dipeptidase. The variable upfield shifts of (Cys–Py_4F_)–Gly perhaps stemmed from environmental changes induced by the cell membrane enzyme of γ-GT.^13^ Although the probe with the lipophilic cation of pyridinium was generally assumed to accumulate in mitochondria, the time-varying ^19^F NMR spectra were found to reflect the whole-cell thiols using the fluorescent wPSP-4F–HEN, a derivative of wPSP-4F, presumably owing to the high concentration in our case (Fig. 3F and S31†).^38^

### Thiol related enzyme activity detection

**Fig. 4:**
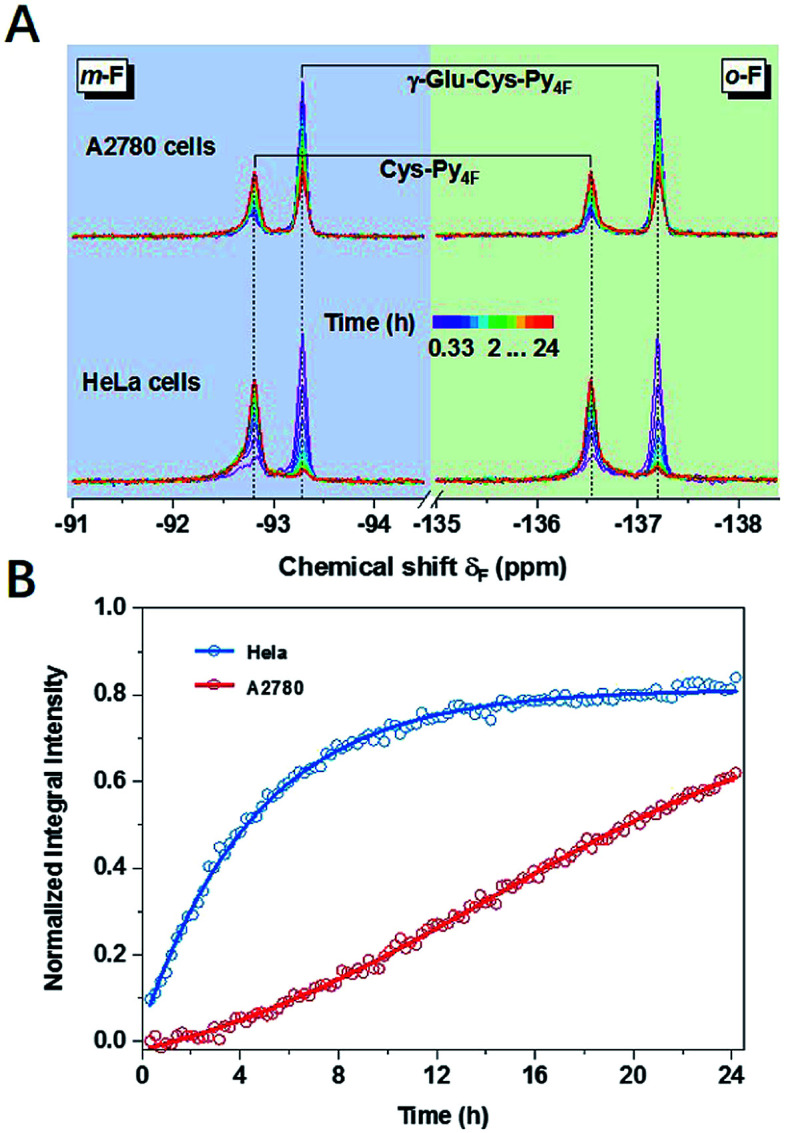
(A) Time-dependent ^19^F NMR spectra of Cys–Py_4F_ after incubating γ-Glu–Cys–Py_4F_ (170 μM) with HeLa or A2780 cells (*ca.* 4 × 10^7^, in 350 μL cell-culture medium, 10% D_2_O, rt) and (B) corresponding time-course of integral intensities.

The concept of thiol discrimination could also be easily extended to detect enzyme activities based on suitable thiol or thiol–Py_4F_ substrates. For example, γ-Glu–Cys–Py_4F_ could be used to detect the activity of the aforementioned γ-GT, which was extensively studied as a diagnostic index for drug-induced liver injury or some types of cancer.^39^ Instead of continuous degradation of GS–Py_4_ with ambiguous signals (1-a and 1-b, Fig. 3E), γ-Glu–Cys–Py_4F_ exhibited a single reliable response towards different concentrations of γ-GT (Fig. S32†). As illustrated, evidently different activities of γ-GT were observed in two cell lines of HeLa and A2780 under the same conditions, which suggested that the probe has the potential to diagnose related diseases (Fig. 4). We believed that this method could easily detect other kinds of enzyme activities by rational fusion of Cys (Cys–Py_4F_) into recognition sequences.^40–42^

## Conclusions

In summary, we have rationally developed a practical platform (PSP-4F and derivatives) that enabled selective and rapid trapping of thiols and outputting characteristic ^19^F NMR signals for easy quantification. Based on the perturbations of ^19^F atoms induced by different electron densities and spatial structures around the sulfhydryl group, the probe discriminated multiple small molecule thiols through unique “2D codes”. The method showed powerful discriminatory ability that can detect up to 20 small molecule thiols simultaneously. The ^19^F NMR platform presented here potentially had wide applications, illustrated by reliable determination of drug content, long-term tracking of biothiols in living cells, and detection of γ-GT activities. We expect that our concept could further realize the monitoring of thiol-related physiological activities for disease diagnosis.

## Conflicts of interest

There are no conflicts to declare.

## Supplementary Material

SC-012-D0SC04664G-s001
