# Supplementary material for: Simultaneous detection of small molecule thiols with a simple 19F NMR platform
Source: Chem Sci. 2020 Nov 13;12(3):1095–100. doi: 10.1039/d0sc04664g (PMC8179020; doi:10.1039/d0sc04664g)
Supplement: SC-012-D0SC04664G-s001 [file SC-012-D0SC04664G-s001.pdf]

Electronic Supplementary Material (ESI) for Chemical Science.
This journal is © The Royal Society of Chemistry 2020

Electronic Supplementary Information

Simultaneous Detection of Small Molecule Thiols with a Simple ^{19}F NMR Platform

Zhaofei Chai,[†a] Qiong Wu,[†ab] Kai Cheng,[a] Xiaoli Liu,[a] Ling Jiang,[ab] Maili Liu,*[ab] and Conggang Li*[ab]

[a] Key Laboratory of Magnetic Resonance in Biological Systems, State Key Laboratory of Magnetic Resonance and Atomic and Molecular Physics, National Center for Magnetic Resonance in Wuhan, Wuhan National Laboratory for Optoelectronics, Wuhan Institute of Physics and Mathematics, Innovation Academy for Precision Measurement Science and Technology, Chinese Academy of Sciences, Wuhan 430071, China

[b] Graduate University of Chinese Academy of Sciences, Beijing 100049, China

[†] These authors contributed equally to this work.

E-mail: ml.liu@wipm.ac.cn (M.Liu); conggangli@wipm.ac.cn (C. Li)

Contents

1 General Information 3
2 Measurement 3
2.1 General procedure for NMR analysis 3
2.2 Reactivities of fluorinated sulfoxides 3
2.3 Sensitivity of fluorinated sulfoxides 4
2.4 Influence of pH values and organic solvent on the reaction rate 4
2.5 Selectivity and discrimination of wPSP-4F 5
2.6 Quantitative detection of thiols 5
2.7 Determination of NAC in pharmaceutical samples 5
2.8 Cell culture and imaging 6
2.9 Determination of thiols in cell lysates 6
2.10 Evaluation of cytotoxicity of wPSP-4F 6
2.11 In-cell NMR 6
2.12 Detection of the activity of γ-GT 7
3 Supplementary Figures 7
4 Synthesis 25
5 Reference 29

1 General Information

All chemical reagents and solvents were purchased from commercial suppliers and used without further purification. ^{1}H NMR, ^{13}C NMR and ^{19}F NMR spectra for compound identification were recorded on Bruker Avance Spectrometers at 600 MHz with a triple-resonance cryogenic probe or at 500 MHz with a double-resonance broadband probe at room temperature. Chemical shifts (δ) were reported relative to the signals of residual solvent $CDCl_3$ (7.26 ppm for ^{1}H, 77.16 ppm for ^{13}C), MeOD-d_4 (3.31 ppm for ^{1}H, 49.00 ppm for ^{13}C) or external standard trifluorotoluene (-63.72 ppm for ^{19}F). All NMR experiments for thiol-sensing were performed at 600 MHz (564 MHz for ^{19}F) and the ^{19}F NMR spectra were calibrated by internal reference of CF_3COONa (TFA, -75.46 ppm). High-resolution mass spectrometry (HRMS) were recorded on the Agilent Technologies 6530 (Q-TOF) equipped with an Agilent HPLC 1200 series. UV-Vis and fluorescence spectra were obtained on the Evolution 220 spectrophotometer and Edinburgh Instruments FS5 fluorescence spectrometer, respectively. Cell images were taken on the Leica TCS SP8 laser-scanning microscope.

2 Measurement

2.1 General procedure for NMR analysis

All spectra were processed and analyzed with Topspin 3.2 (Bruker). As the integral intensity and concentration were linearly dependent, the consumption rate of sulfoxides could be determined by detecting the corresponding integral intensity over time. In our study, the initial integral intensity of sulfoxide (I_0) was set to 1 unless otherwise noted. The integral intensity of remaining sulfoxide (I_t) and newly-formed thioether (I_t^P) upon addition of thiols at time *t* was calculated as:

$$I_t = \frac{\sum i_s}{\sum i_p + \sum i_s} \text{ or } I_t^P = \frac{\sum i_p}{\sum i_p + \sum i_s} \qquad \textbf{(1)}$$

where i_s and i_p were the total integral intensities of sulfoxide and product in each spectrum, respectively. In each experiment, the spectra were shown at the beginning and regular intervals for clarity.

2.2 Reactivities of fluorinated sulfoxides

Cys was chosen as a typical thiol to compare the reaction rates of PSP, PSP-*o*F, PSP-*o*2F, PSP-*m*2F, PSBN-*o*2F, PSP-4F, and PSBN-4F with sulfhydryl group. The experiments were conducted by mixing Cys (10.0 mM) with sulfoxides (1.0 mM) in PB (100 mM, pH=7.40, 10 % D_2O, 30% AN) at room temperature and detected by ^{19}F NMR or ^{1}H NMR for 1 h. Note that a period of time (40-60 s) was required for sample preparation and program

setup, and the spectra within this range could not be determined. For PSP, only the peaks at aromatic region (9.2-7.2 ppm) were considered in ^{1}H NMR spectra due to the interference signals of solvent and excessive Cys. In order to verify the rearrangement of PSPBN-4F upon addition of Cys, the ^{19}F and ^{1}H NMR spectra after the addition of NAC (N-acetyl-L-cysteine) were recorded under same conditions for comparison (100 mM, pH=7.40, 30% AN-d_3). **(Figure S1-S8)**

2.3 Sensitivity of fluorinated sulfoxides

The ^{19}F NMR spectra were collected after incubating the sulfoxides (1.0 mM) with three thiols (10.0 mM) in PB (100 mM, pH=7.40, 10 % D_2O, 30% AN) at room temperature for 6 h. The spectra were normalized and calibrated by the internal reference of TFA (not shown). **(Figure S9)**

2.4 Influence of pH values and organic solvent on the reaction rate

The reactions at different pH values were conducted in acetate buffer (100 mM, 10 % D_2O, 30% AN, pH = 4.0 and 5.0) or PB (100 mM, pH=7.40, 10 % D_2O, 30% AN, pH = 6.0, 7.0 and 8.0) at room temperature. While the solution with a different fraction of organic solvent was prepared with AN and Tris buffer for better miscibility (100 mM, pH=7.40, 10 % D_2O, 28-67% AN). In the experiments, the solution of PSP-4F (1.0 mM) and Cys (2.5 mM) in the tube was shaken for 5-10 seconds and placed in the NMR instrument immediately with a delay of 40-60 s. The reactions were monitored for different times depending on the conversion efficiency (> 0.95) except the reaction at pH 4.0, which was detected for 24 h due to slow kinetics. In data processing, the integral intensities of PSP-4F and Cys-Py_{4F} were calculated with equation **(1)**. The nucleophilic attack of -SH on sulfones **(2)** was extrapolated to the second-order reaction, which was determined as follows.

$$\text{PSP-4F} + \text{Cys} \rightarrow \text{Cys-Py}_{4F} + \text{PhSO}_2\text{H} \qquad \textbf{(2)}$$

If x reactant (PSP-4F or Cys) was consumed at time t, the remaining reactant ($[\text{reactant}]_t$) could be calculated from the initial one ($[\text{reactant}]_0$) as: $[\text{reactant}]_t = [\text{reactant}]_0 - x$. Thus, the reaction rate (r) and the second-order rate constant (k_2) could be defined as follows.

$$r = \frac{dx}{dt} = k_2[PSP\text{ - }4F]_t[Cys]_t = k_2([PSP\text{ - }4F]_0 - x)([Cys]_0 - x) \qquad \textbf{(3)}$$

$$\int \frac{dx}{([PSP\text{ - }4F]_0 - x)([Cys]_0 - x)} = \int k_2 dt \qquad \textbf{(4)}$$

$$\frac{1}{[PSP\text{ - }4F]_0 - [Cys]_0} ln \frac{[PSP\text{ - }4F]_0 - x}{[Cys]_0 - x} = k_2 t + C \quad \text{(C: constant)} \qquad \textbf{(5)}$$

Here, the concentration was linearly correlated with integral intensity and the initial integral intensity of sulfoxide (I_0) was set to 1 (corresponding to 1.0 mM probe). According to the stoichiometric coefficients **(2)**, the

consumption of PSP-4F could also be replaced with Cys-Py$_{4F}$ (I_t^P). Additionally, if the initial Cys was n times larger than PSP-4F ($[Cys]_0 = n[PSP\text{-}4F]_0$), the equation (5) can be expressed as follows.

$$\frac{1}{I_0 - nI_0} ln \frac{I_0 - I_t^P}{nI_0 - I_t^P} = y = k_2 t + C \quad \textbf{(6)}$$

Based on the following experiments, the calculated y was almost linearly proportional to t ($R^2 > 0.97$), which validated our hypothesis's rationality. The calculated k_2 in each condition was given in the figure. The k_2 values for wPSP-4F and wPSP-4F-HEN were determined with the same procedures. **(Figure S10-S13, S31)**

2.5 Selectivity and discrimination of wPSP-4F

Generally, analytes (10 mM) were incubated with wPSP-4F (100 μM) in PB (100 mM, pH=7.40, 10 % D_2O, rt) for 1 h and detected with ^{19}F NMR spectra at room temperature. D-PEN was detected after 3 h incubation with wPSP-4F at room temperature. 6-MP and MMI were incubated with wPSP-4F for 72 h at 37 ^{o}C and then detected at room temperature. **(Figure S14 and S19-22)**

2.6 Quantitative detection of thiols

The quantitative detection of thiols was illustrated with wPSP-4F and GSH as a typical example. In the titration, wPSP-4F and GSH were incubated for about 20 min at room temperature and detected with ^{19}F NMR spectra. Note that the integral intensity of TFA was set to 1 in this section. The integral intensities of two distinct peaks with a wide distribution ($\Delta\delta > 50$ ppm) were nearly equal in every measurement, which confirmed the full activation of environmentally different ^{19}F atoms in our experiment.

A reported approach was used to determine the LOD and LOQ[1], as: LOD = $y_{blind} + 3S_{blind}$, LOQ = $y_{blind} + 9S_{blind}$, where y_{blind} and S_{blind} were the mean value and standard deviation of the noise, respectively. An illustration was given by the detection and quantification of GSH in a diluted solution. **(Figure S15 and S16)**

2.7 Determination of NAC in pharmaceutical samples

The standard NAC and commercial acetylcysteine effervescent tablets (Zhejiang Jinhua Conba Bio-Pharm. Co., LTD) (average weight of tablets: 1792 mg; NAC content: 600 mg) were dissolved in PB (100 mM, pH=7.40) to obtain *ca.* 1 mM stock solution. In the ^{19}F NMR measurement, the diluted solution (100 μM) was mixed with wPSP-4F (*ca.* 400 μM) in PB (100 mM, pH=7.40, 10 % D_2O, rt) for 20 min and tested. The HPLC measurement was conducted with a modified procedure (column: ODS; eluent: H_2O containing 5% CH_3CN and 1% HCOOH; flow rate:1.0 mL min^{-1}; detection wavelength: 215 nm; sample volume: 50 μL).[2] The typical spectra were shown in

Figure S21 and S22. The result represented the mean value of three independent experiments. **(Figure S17 and S18)**

2.8 Cell culture and imaging

Cells were grown at 37 ^{o}C in 5%/95% (v/v) CO_2/humidified air incubators in high-glucose Dulbecco's modified Eagle's medium (DMEM) supplemented with 10% fetal bovine serum (FBS) and 1% penicillin–streptomycin. For fluorescence imaging, HeLa cells were pretreated with wPSP-4F-HEN (2 mM) and MitoTracker (0.5 μM) successively at 37 ^{o}C for 30 min in each step, and then washed 3 times with a PBS solution. Confocal images were obtained using an excitation wavelength of 488 nm.

2.9 Determination of thiols in cell lysates

Lysates were prepared with Hela cells by ultrasonic disrupter at 0 ^{o}C, then freeze-dried and stored at -80 ^{o}C. The dried lysates, aiming to eliminate the influence of cell volume and cell counting results, were resuspended in PB (100 mM, pH=7.40) and dialyzed with 3K cut-off membrane at 4 ^{o}C. The solution passed through the membrane was used to determine the small molecule thiols, while the residue was washed 3 times with PB again and used to measure the influence of free cysteine residues in proteins. **(Figure S23)**

2.10 Evaluation of cytotoxicity of wPSP-4F

HeLa cells (*ca.* 1×10^4) were plated into 96 well-plate and incubated for 24 h. After the medium was removed, the cells were incubated with different concentrations of wPSP-4F in DMEM medium containing 10% FBS, and 1% penicillin–streptomycin for 24 h. Then, Cell viability was measured by MTT methods (KeyGEN BioTECH). **(Figure S25)**

2.11 In-cell NMR

Cells were detached from culture dishes with trypsin/EDTA (0.25 %/ 0.02 %) for about 3 min at 37 ^{o}C, and counted on a haemocytometer. Approximate 4×10^7 cells were typically used as the starting material. The cells were collected by centrifugation at 200 × g for about 10 min at rt and resuspended in the culture medium (90 mM glucose, 5 mM HEPES in DMEM) containing the probe and 5% D_2O for NMR experiment. The stock solutions of GS-Py_{4F}, Cys(-Py_{4F})-Gly and γ-Glu-Cys(-Py_{4F}) were prepared by adding wPSP-4F (2.0 mM) into corresponding thiols (3.0 mM) in culture medium at 25 ^{o}C for 20 min (checked by ^{19}F NMR spectra) and used without further purification. Note that the initial state (15-20 min) of in-cell thiols could not be obtained due to the delay for sample preparation and program setup in this process. **(Figure S24, S26-30)**

2.12 Detection of the activity of *γ*-GT

The activity of *γ*-GT was measured with the same procedure of thiol detection. Note that the commercial product of *γ*-Glu-Cys (Shanghai Aladdin Biochemical Technology Co., Ltd, China) contained a portion of Cys and the contents of two species were determined by the ^{19}F NMR spectra in dilute solutions. The amount of original Cys was subtracted from the total integral intensity when calculating the newly formed one. **(Figure S32)**

3 Supplementary Figures

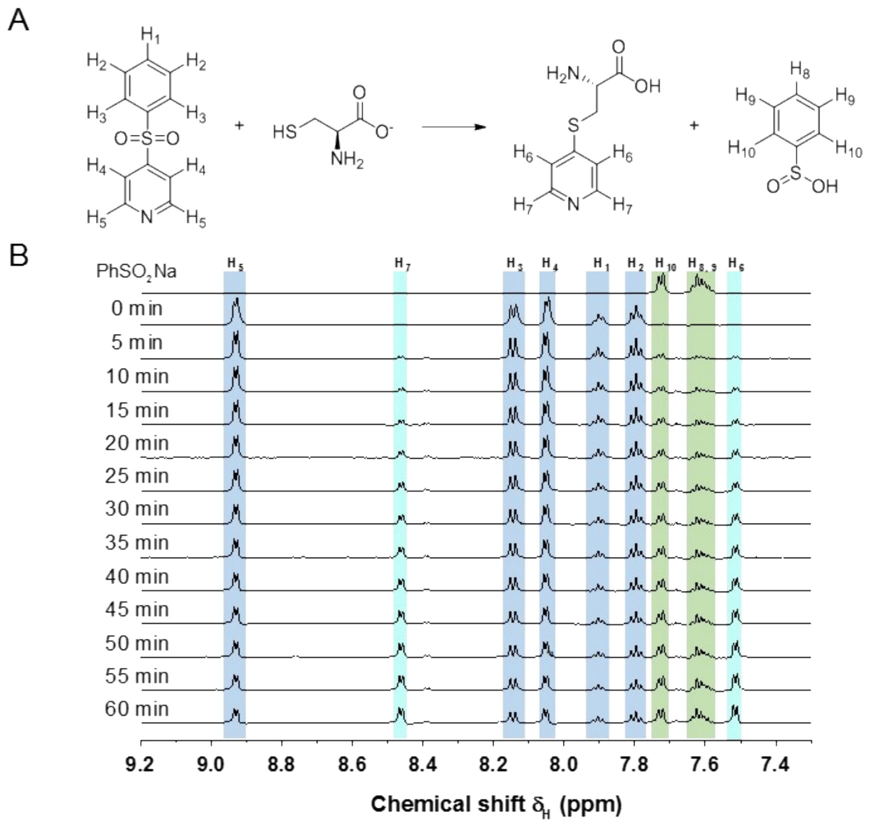

Figure S1 (A) Reaction equation of PSP with Cys and corresponding atom numbers. (B) Time-dependent ^{1}H NMR spectra (aromatic region) of PSP upon the addition of Cys.

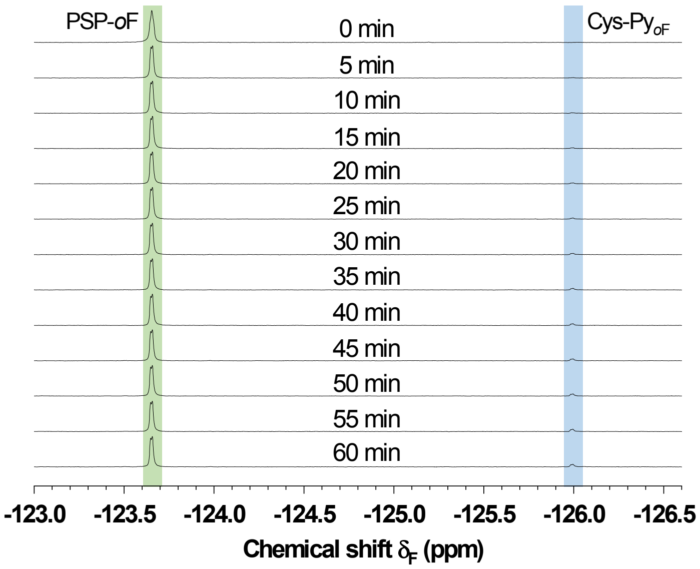

Figure S2 Time-dependent ^{19}F NMR spectra of PSP-*o*F upon the addition of Cys.

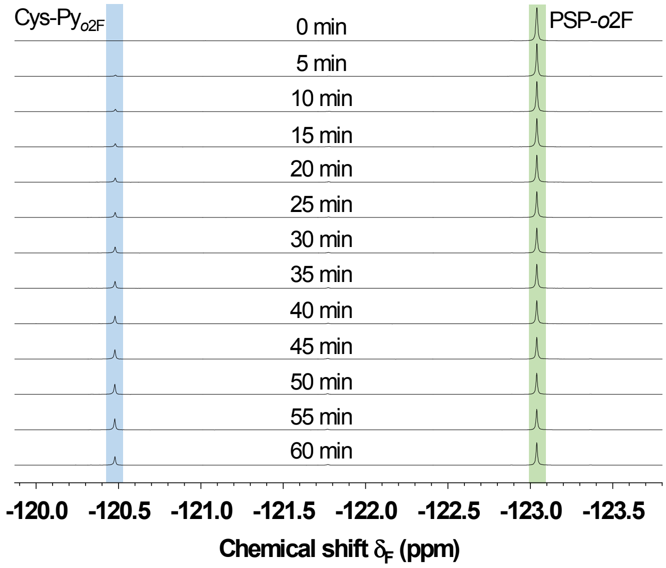

Figure S3 Time-dependent ^{19}F NMR spectra of PSP-*o*2F upon the addition of Cys.

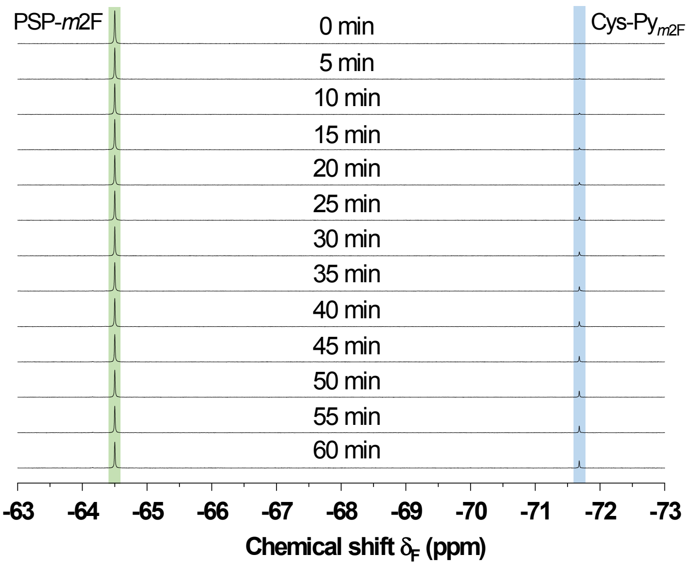

Figure S4 Time-dependent ^{19}F NMR spectra of PSP-*m*2F upon the addition of Cys.

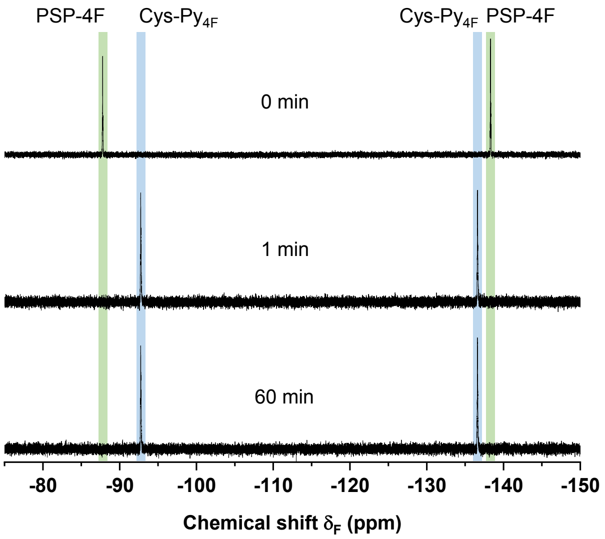

Figure S5 Time-dependent ^{19}F NMR spectra of PSP-4F upon the addition of Cys.

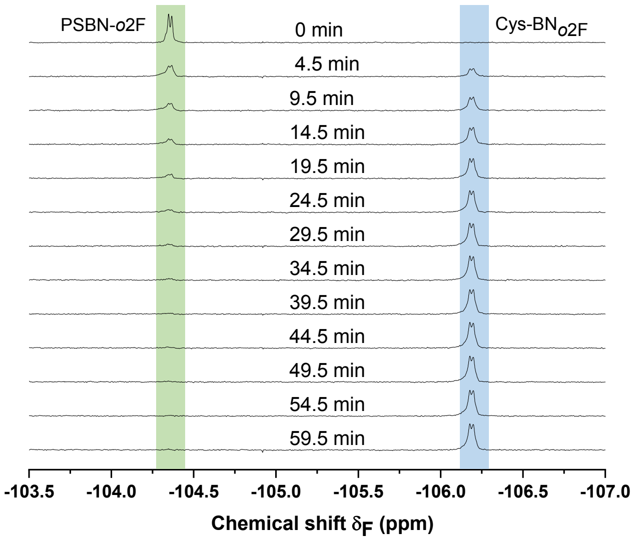

Figure S6 Time-dependent ^{19}F NMR spectra of PSBN-*o*2F with the addition of Cys.

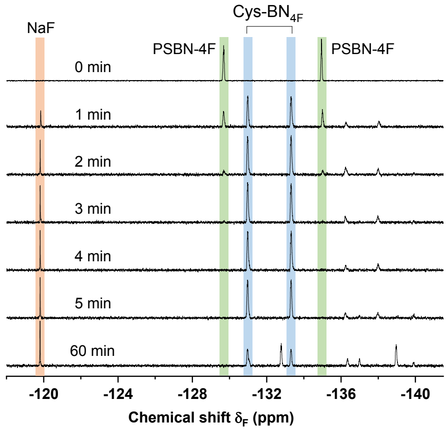

Figure S7 Time-dependent ^{19}F NMR spectra of PSBN-4F with the addition of Cys. Only PSBN-4F and the initial main product of Cys-BN_{4F} was marked.

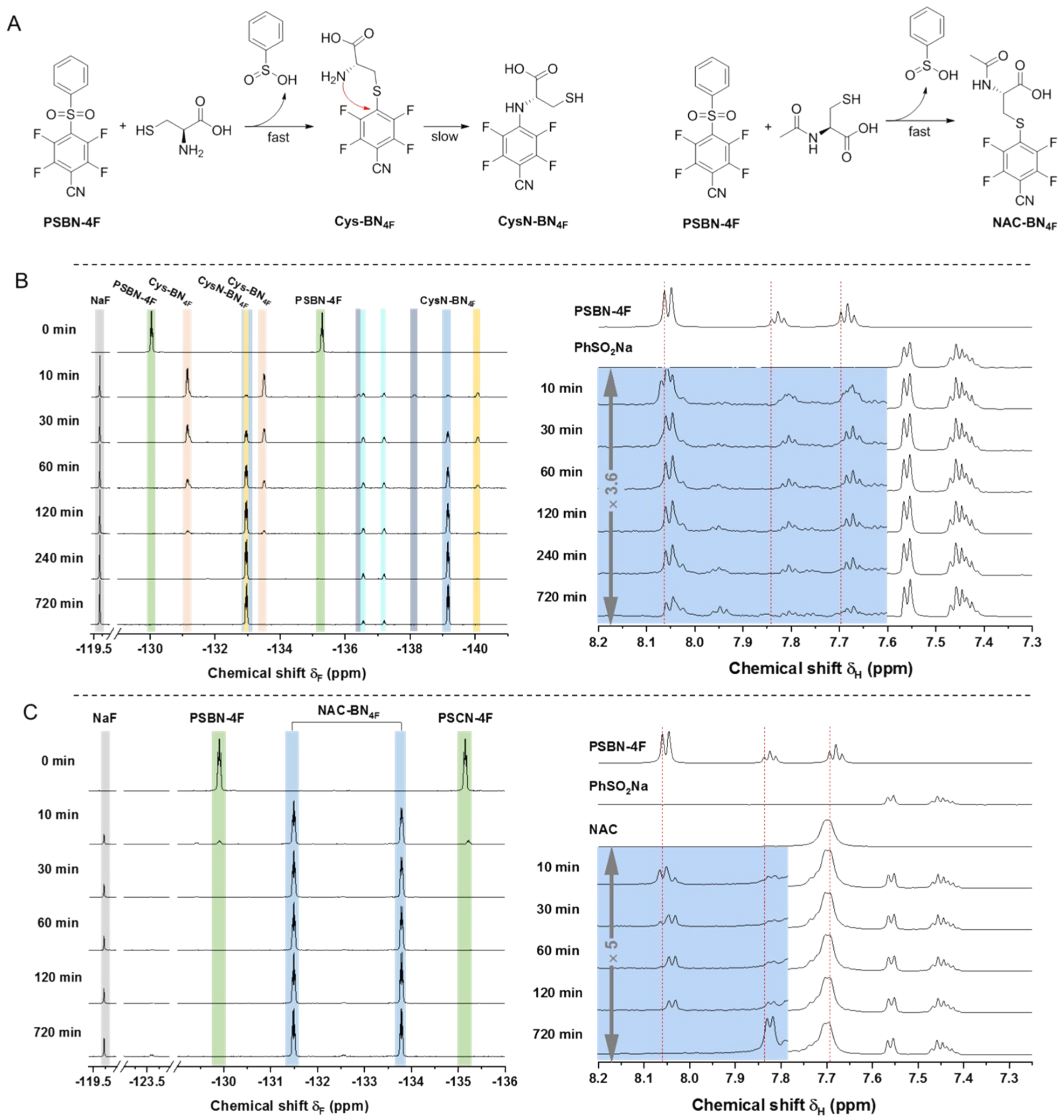

Figure S8 (A) Main reaction paths of PSBN-4F with Cys and NAC. (B) Time-dependent ^{19}F NMR (left) and ^{1}H NMR (right) spectra (aromatic region) of PSBN-4F upon the addition of Cys. Peaks with the same integral intensities were marked with the same color in the ^{19}F NMR spectra. (C) Time-dependent ^{19}F NMR (left) and ^{1}H NMR (right) spectra (aromatic region) of PSBN-4F upon the addition of NAC.

As shown in ^{19}F NMR spectra, the PSBN-4F in two reactions was almost depleted within 10 min, and two main peaks were observed with similar chemical shifts (Cys: -131.15 and -133.51 ppm; NAC: -131.49 and -133.79 ppm). After 240 min, two main peaks of Cys/PSBN-4F moved to -132.96 and -139.16 ppm, while those of NAC/PSBN-4F without α-primary amine did not show any change. Besides, the ^{1}H NMR of two reactions displayed the peaks of $PhSO_2Na$, which was one of the main products after nucleophilic substitution of sulfoxides. So, the change of two main peaks in ^{19}F NMR spectra of Cys/PSBN-4F resulted from the Cys-induced S_NAr substitution-rearrangement reaction, which has been well studied in the literature.[3] However, many small peaks were also found

in the ^{19}F NMR spectra of two reactions, especially for Cys.[4, 5] Due to the emergence of NaF, the other nucleophile substitutions (PSBN-nF-Cys, n = 2, 3) were supposed to occur between the F atoms and sulfhydryl group. The ^{1}H NMR also verified the presence of F-substituted sulfoxides, which showed much lower integral intensity and less changeable chemical shift compared with those of $PhSO_2Na$. Nevertheless, it seemed that the initial S-substituted products also gradually changed into N-substituted ones. Eventually, only a set of small peaks was found. It was hard to determine the structure due to the low contents.

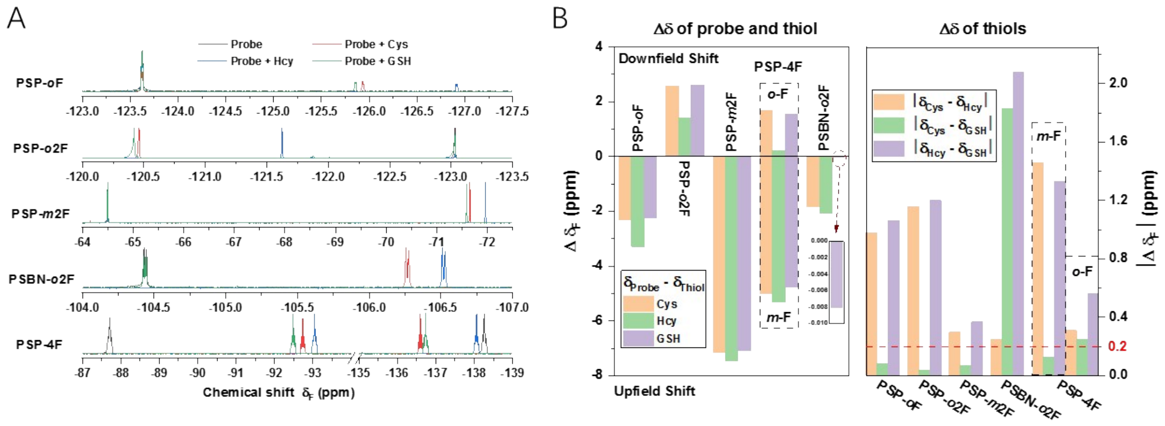

Figure S9 (A) ^{19}F NMR spectra of fluorinated sulfoxides upon the addition of Cys, Hcy, and GSH. (B) Differences in chemical shifts between sulfones and thiols (left) and thiols themselves (right).

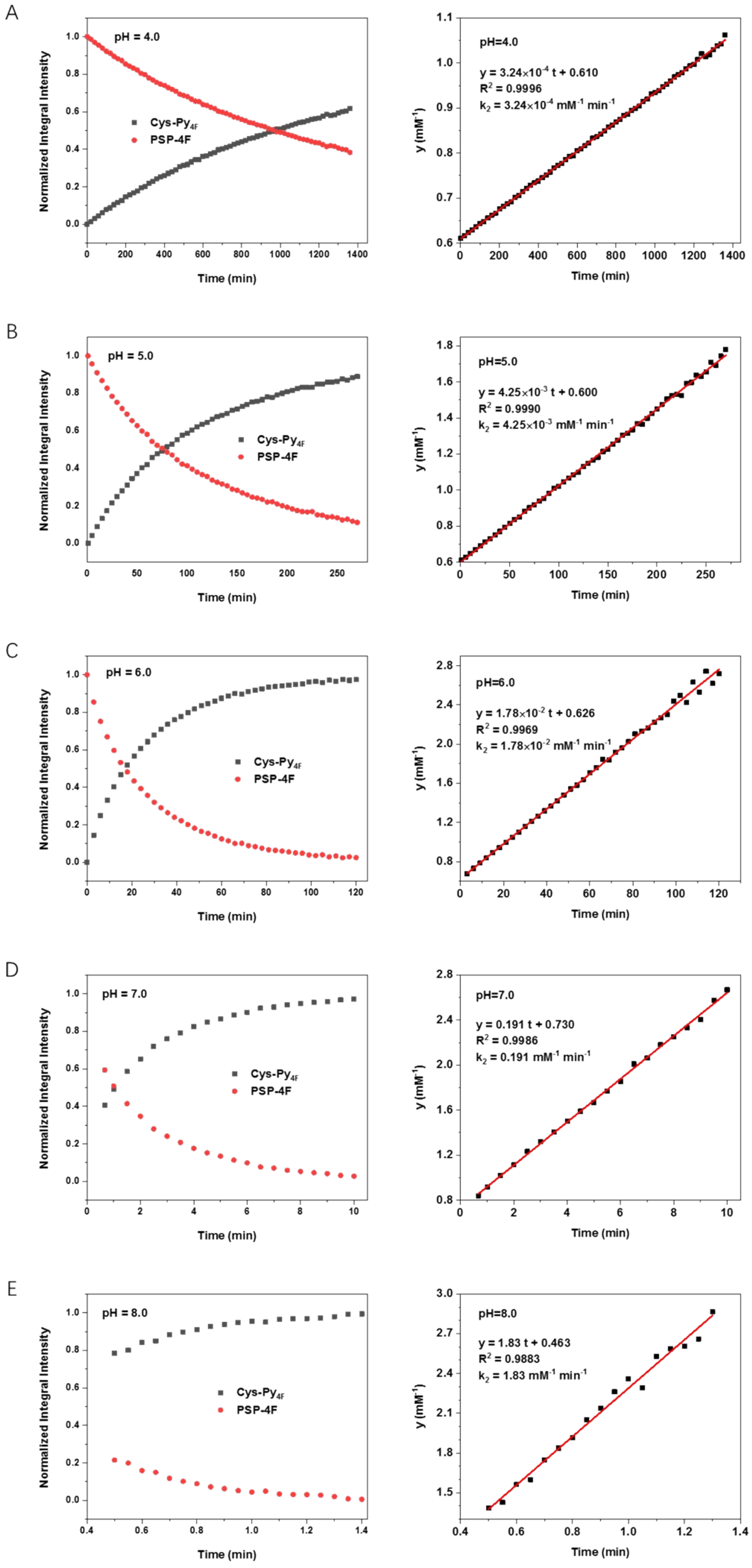

Figure S10 Time-dependent integral intensities of Cys-Py$_{4F}$ and PSP-4F (left) and *y-t* curves (right) at different pH values.

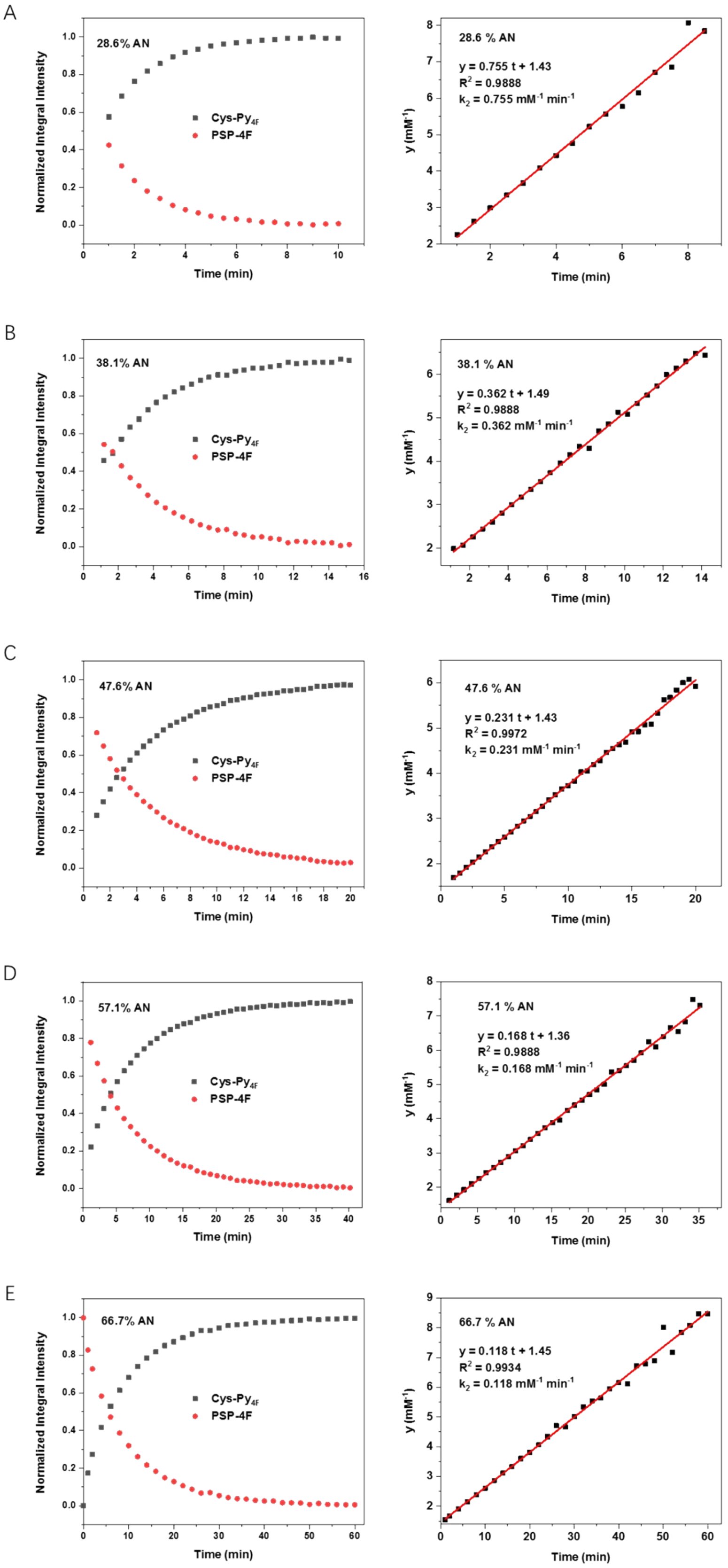

Figure S11 Time-dependent integral intensities of Cys-Py_{4F} and PSP-4F (left) and *y-t* curves (right) with the different volume fraction of AN.

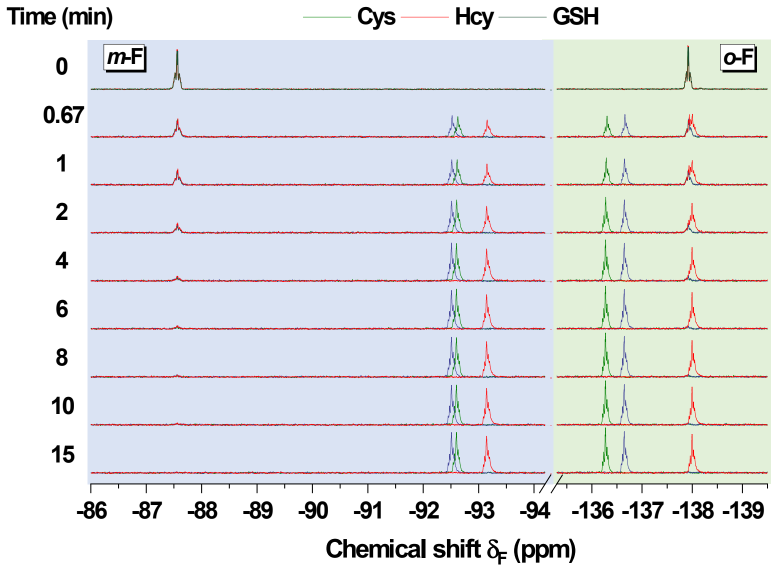

Figure S12 Time-dependent ^{19}F NMR spectra of wPSP-4F (1.0 mM) toward Cys, Hcy and GSH (1.5 mM) in PB (100 mM, pH=7.40, 10% D_2O, rt).

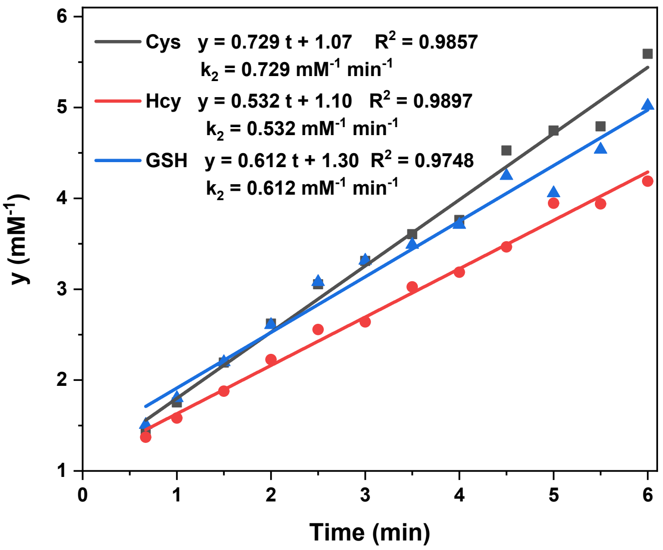

Figure S13 *Y-t* curves between wPSP-4F (1.0 mM) and Cys, Hcy and GSH (1.5 mM) in PB (100 mM, pH=7.40, 10% D_2O, rt). Note that only *m*-F was considered for Hcy.

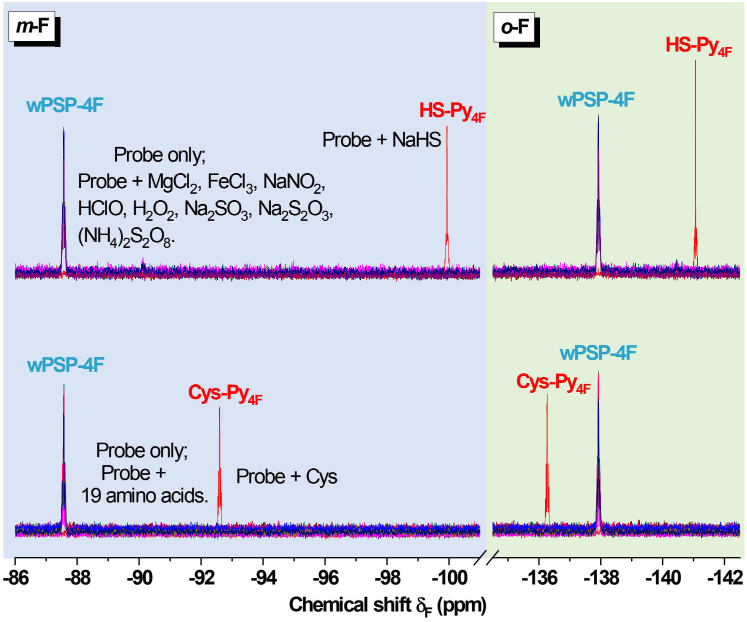

Figure S14 ^{19}F NMR spectra of wPSP-4F (100 μM) upon addition of (top) typical inorganic ions (10 mM) and (bottom) 20 amino acids (10 mM) in PB (100 mM, pH=7.40, 10 % D_2O, rt).

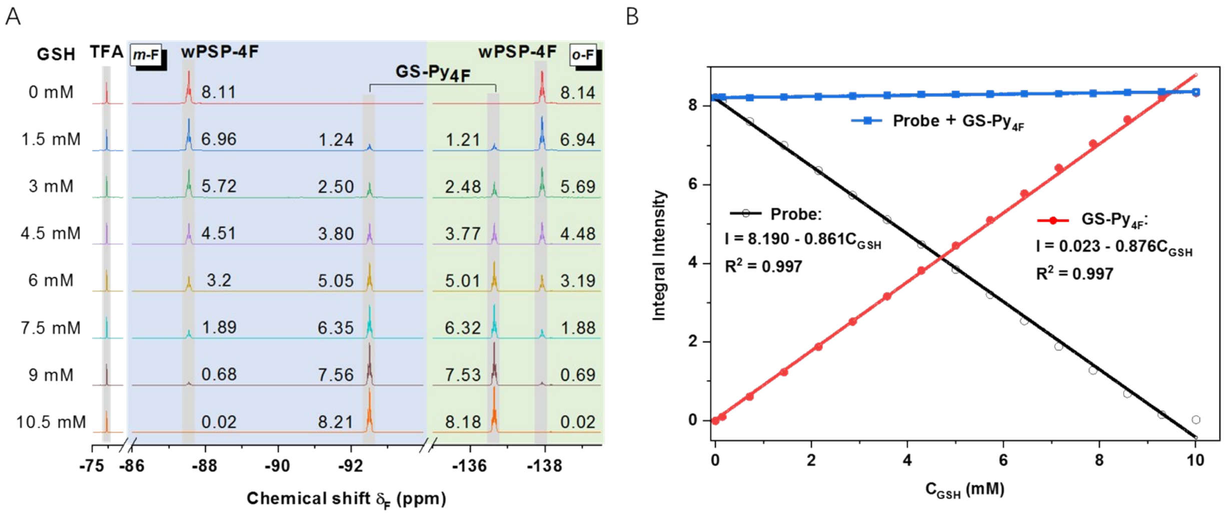

Figure S15 (A) ^{19}F NMR spectra of wPSP-4F (10.0 mM) upon addition of GSH (0-10.5 mM) in PB (100 mM, pH=7.40, 10 % D_2O, 25 °C) and (B) dose–response curves of probe and adduct versus GSH. Each ^{19}F NMR spectrum was taken 20 min after the addition of GSH. The integral intensity of TFA serves as a benchmark (internal reference, normalized to 1), and other relative integral intensities were labeled next to the peaks.

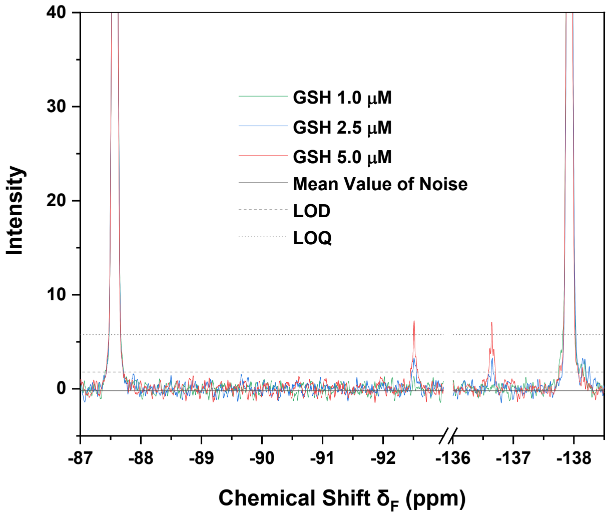

Figure S16 ^{19}F NMR spectra of wPSP-4F (100 μM) upon addition of GSH in PB (100 mM, pH=7.40, 10 % D_2O). The noise range was set from -89 to -91 ppm. LOD and LOQ of GSH in dilute solutions were determined to be lower than 2.5 and 5.0 μM, respectively, at 564 MHz, 512 scans (13 min 4 s) at room temperature.

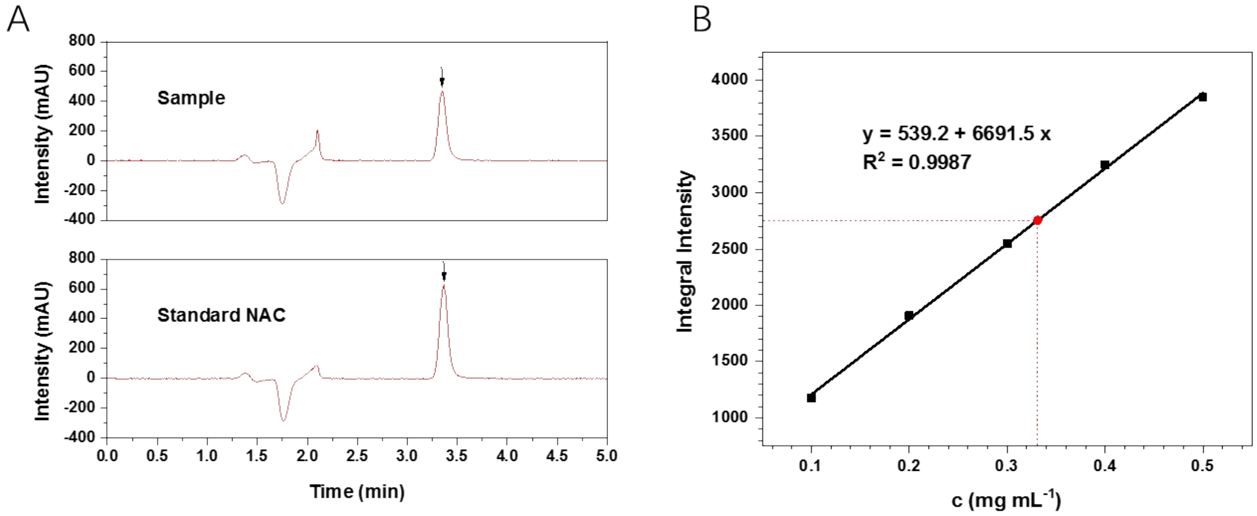

Figure S17 (A) HPLC traces of standard NAC and NAC effervescent tablets (drug). (B) Standard curve of NAC. The red spot represented the integral intensity of drug in the measurement.

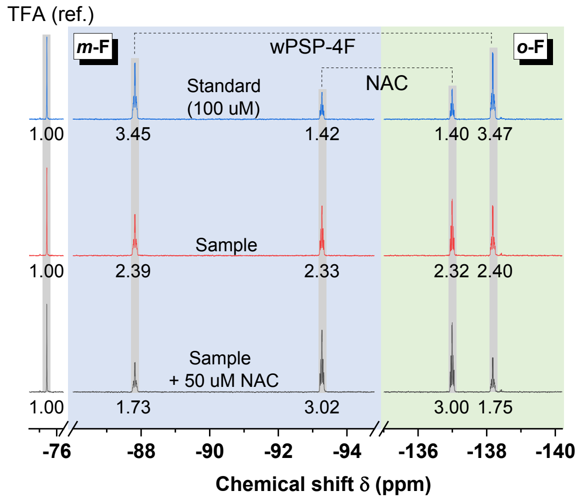

Figure S18 ^{19}F NMR spectra of wPSP-4F upon addition of standard NAC and NAC effervescent tablets (drug) in PB (100 mM, pH=7.40, 10% D_2O).

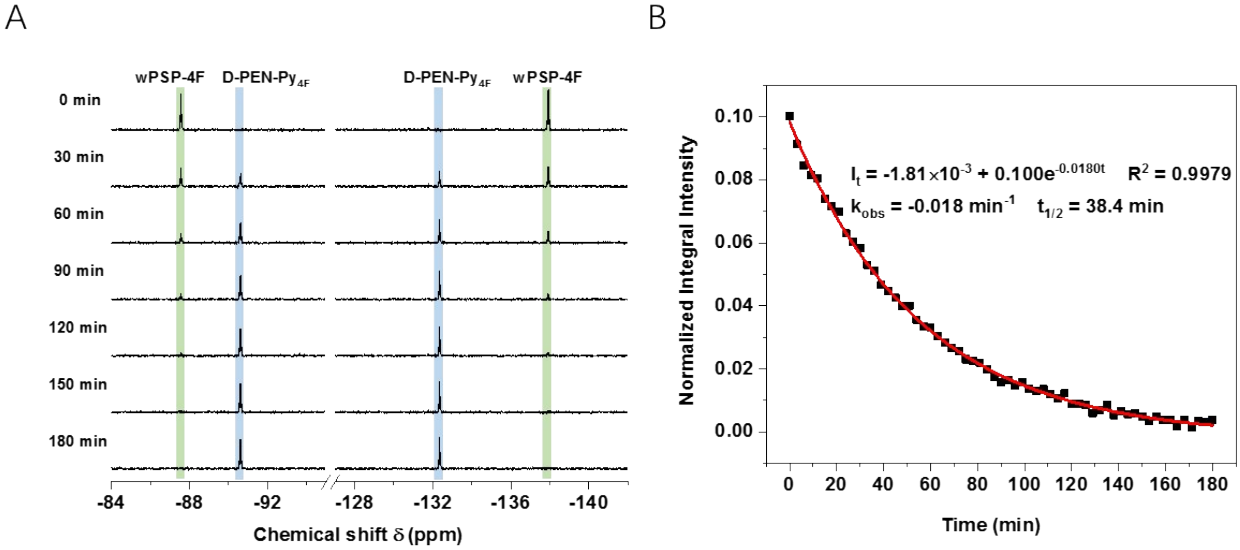

Figure S19 (A) Time-dependent ^{19}F NMR spectra of wPSP-4F (0.1 mM) upon addition of D-PEN (10 mM) and (B) corresponding integral intensity decay curve in PB (100 mM, pH=7.40, 10 % D_2O, rt). The total integral intensities of each spectrum were normalized to 0.1 (corresponding to the initial concentration of wPSP-4F) and the data were fitted with the single exponential function due to the excessive D-PEN (>> 40 equiv), which gave the pseudo-first-order rate (k_{obs}) and half-life ($t_{1/2}$).

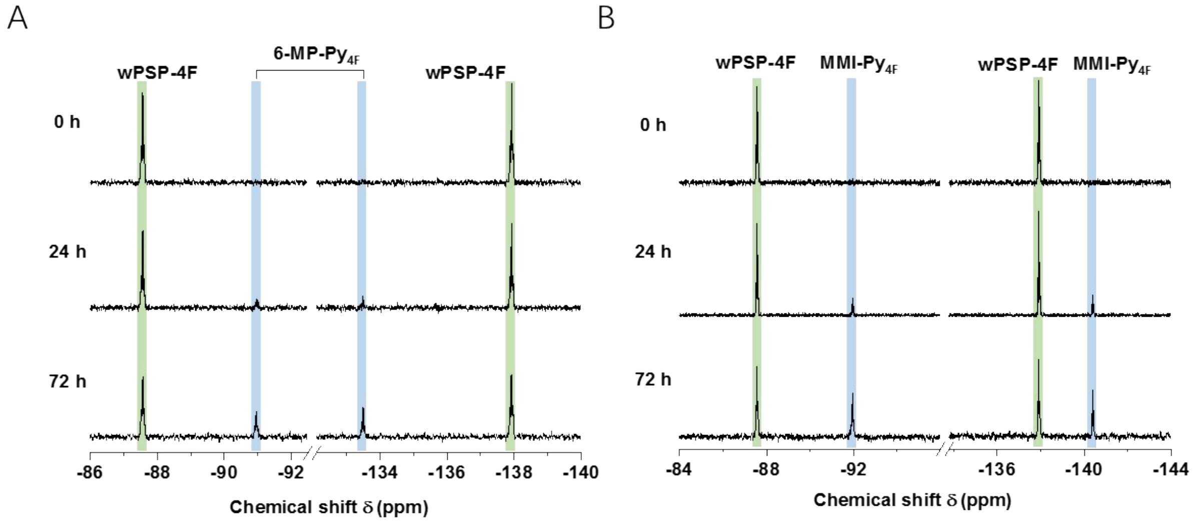

Figure S20 Time-dependent ^{19}F NMR spectra of wPSP-4F (100 μM) upon addition of (A) 6-MP (saturated solution) and (B) MMI (10 mM) in PB (100 mM, pH=7.40, 10 % D_2O, rt). Note that a saturated solution of 6-MP was used due to its limited solubility. According to their integral intensities, about 27% and 35% wPSP-4F reacted with 6-MP and MMI, respectively. The kinetics were not determined.

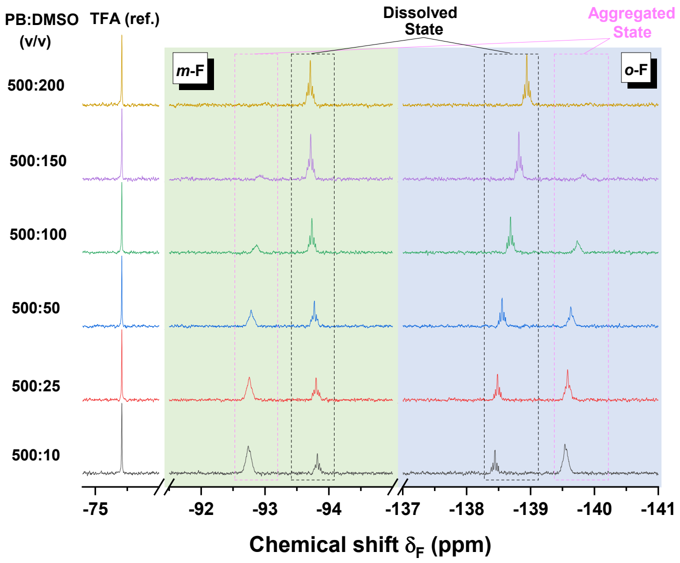

Figure S21 ^{19}F NMR spectra of wPSP-4F (100 μM) upon addition of n-BuSH (10 mM) in a mixture solvent of PB (initial condition: 100 mM, pH=7.40, 10 % D_2O) and DMSO at room temperature. The peak positions were calibrated by the internal reference of TFA. With the increase of DMSO, the aggregate was dissolved and the spectra displayed one set of peaks. This phenomenon was also observed with 1-BuSH, 2-BuSH and t-BuSH.

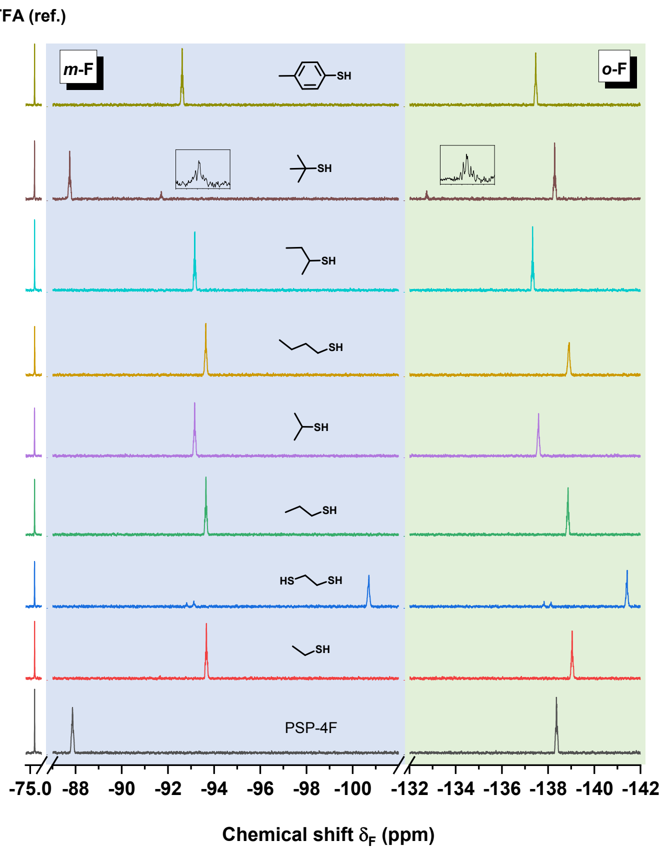

Figure S22 ^{19}F NMR spectra of PSP-4F (100 μM) upon addition of lipophilic thiols (10 mM) in PB (100 mM, pH=7.40, 10% D_2O, 30% AN, rt). The reactions were incubated at room temperature for 1 h and the peak positions and intensities were calibrated by internal reference of TFA.

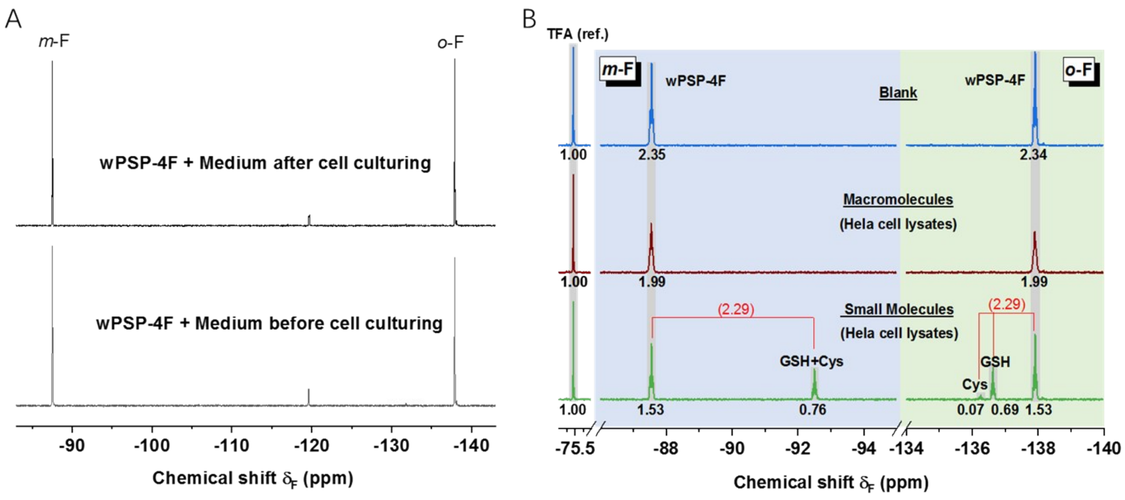

Figure S23 (A) ^{19}F NMR spectra of wPSP-4F upon addition of cell-culture medium (medium, 400 uL; Probe, 5 mM in PBS, 50 μL; D_2O, 50 μL). Note that the small peak located around 119.67 ppm was ascribed to the free F^-

originated from impurities of our product, which was also observed in other experiments (not shown in every spectrum). (B) ^{19}F NMR spectra of the probe (500 μM) with the addition of different components of Hela cell lysates (6 mg/mL) in PB (100 mM, pH=7.40, 10 % D_2O, rt) after 20 min. The numbers represented integral intensities. Top: Blank; Middle: macromolecules after centrifugal separation and dialysis; Bottom: the remaining solution containing small molecules.

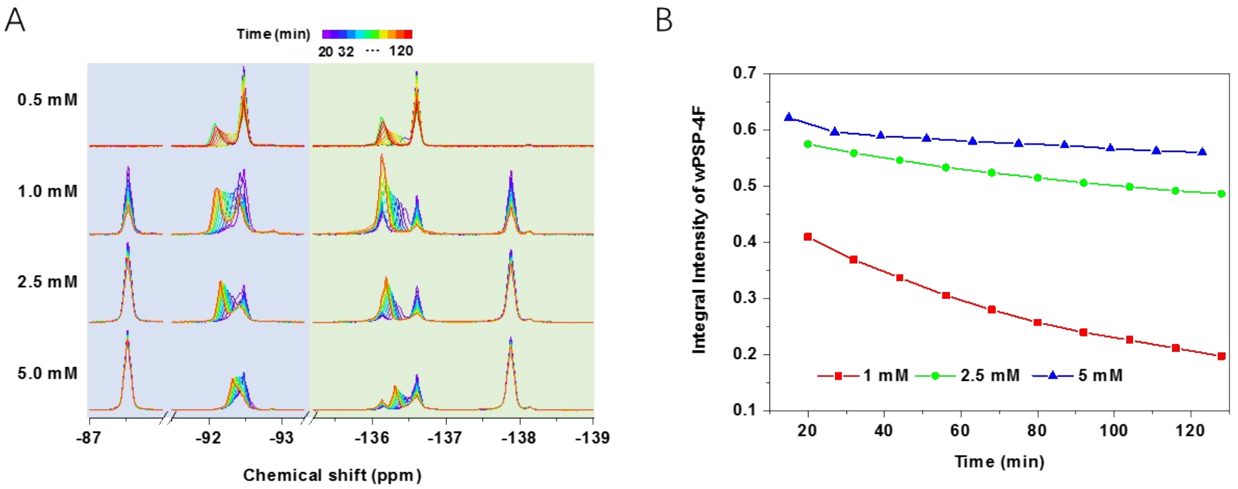

Figure S24 (A) Time-dependent ^{19}F NMR spectra of the probe toward thiols in Hela cells (*ca.* 4 × 10^7, in 400 μL cell-culture medium, 10% D_2O, rt) within 2 h and (B) corresponding integral intensities of wPSP-4F.

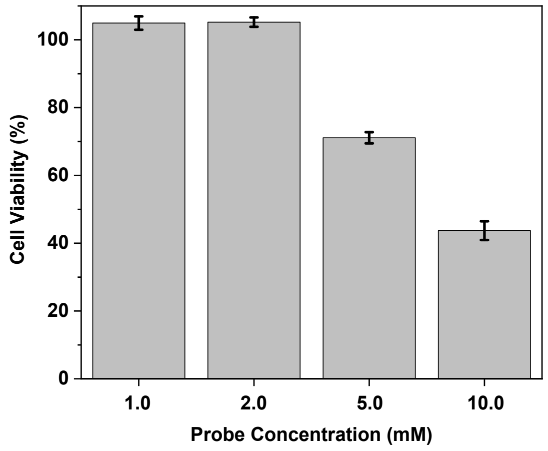

Figure S25 Viability test of wPSP-4F-loaded Hela cells. Cell viability was evaluated 24 hours after probe loading. Error bars represented standard deviation (n = 12).

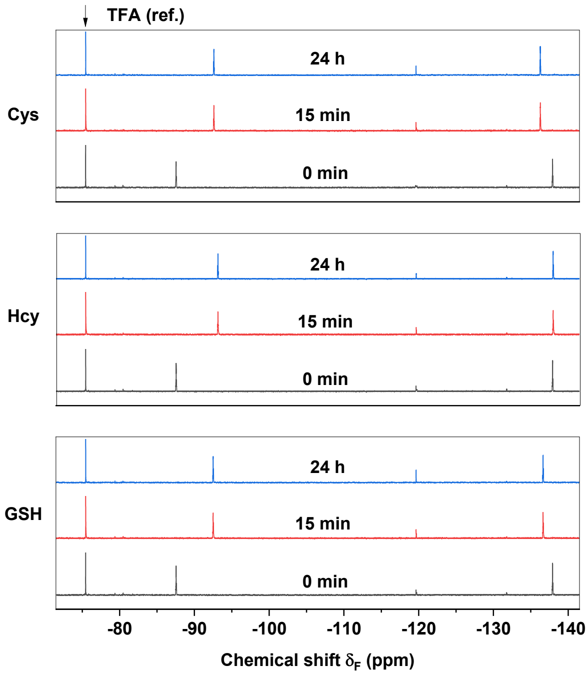

Figure S26 ^{19}F NMR spectra of wPSP-4F (1 mM) with the addition of Cys, Hcy, and GSH (1.5 mM) in PB (100 mM, pH=7.40, 10 % D_2O) at different time when exposed to air at room temperature.

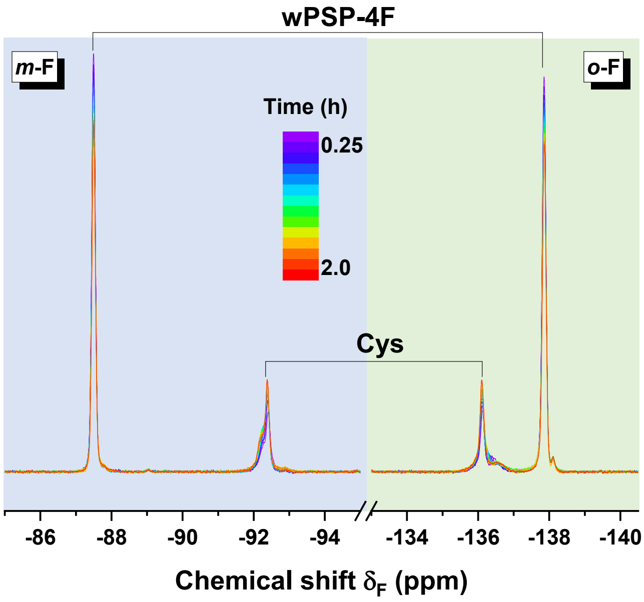

Figure S27 (A) Time-dependent ^{19}F NMR spectra of wPSP-4F (2.0 mM) toward thiols in Hela cells (*ca.* 4 × 10^7, in 400 µL cell-culture medium, 10% D_2O, rt) after being treated with BSO (500 µM) for 24 h.

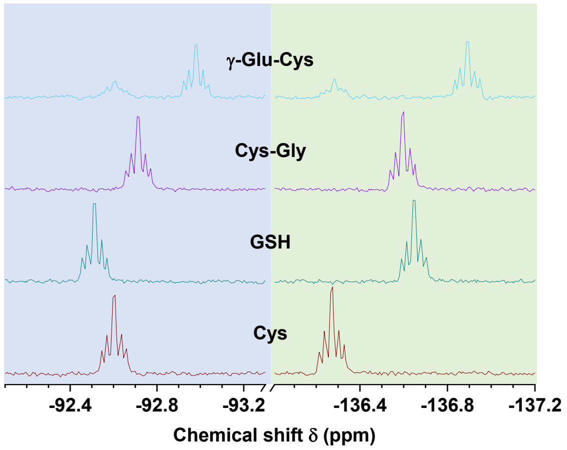

Figure S28 ^{19}F NMR spectra of wPSP-4F (100 μM) upon addition of Cys, GSH, Cys-Gly, and γ-Glu-Cys (10.0 mM) in PB (100 mM, pH=7.40, 10 % D_2O, rt). Note that the commercial product of *γ*-Glu-Cys contained a portion of Cys.

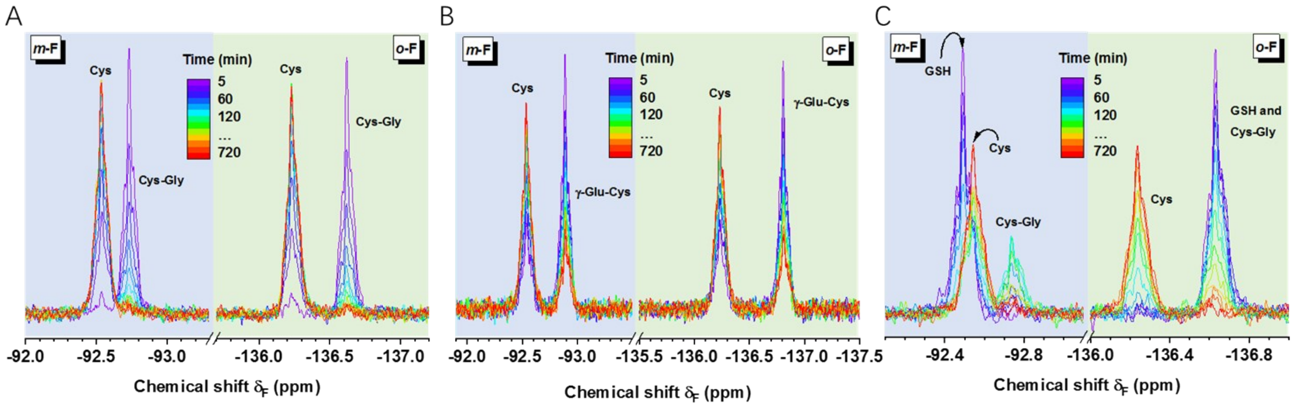

Figure S29 Time-dependent ^{19}F NMR spectra of (A) GS-Py_{4F}, (B) Cys(-Py_{4F})-Gly and (C) *γ*-Glu-Cys(-Py_{4F}) in the presence of fresh cell lysates at 25 °C. The lysates were prepared with Hela cells (*ca.* 4×10^7, in 400 μL cell-culture medium) by ultrasonic disrupter at 0 °C. Each sample contained 270 μL adduct (0.15 mM), 100 μL lysates and 30 μL D_2O. The integral intensities in different degradations were calculated according to eq **(1)**. Note that the commercial product of *γ*-Glu-Cys contained a portion of Cys and the amounts have been subtracted from the total integral intensity when calculating the newly formed one. For GSH, i(Cys) = $2 \times i_{oF}$(Cys) = $2 \times i_{-136.17 \sim -136.37\ \mathrm{ppm}}$, i[Cys(-Py_{4F})-Gly] = $2 \times i_{oF}$[Cys(-Py_{4F})-Gly] = $2 \times i_{-92.68 \sim -92.91\ \mathrm{ppm}}$.

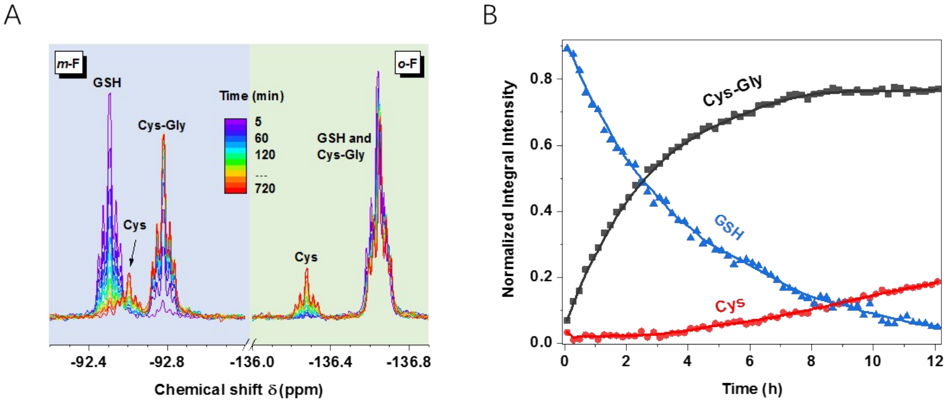

Figure S30 (A) Time-dependent ^{19}F NMR spectra of GS-Py$_{4F}$ (100 μM) in the presence of γ-GT (500 U/L) in PB (100 mM, pH=7.40, 10 % D$_2$O, 25 °C) and (B) corresponding integral intensities. The intensities were calculated as: i(Cys) = 2 × i_{oF}(Cys) = 2 × $i_{-136.17\sim-136.37\ \mathrm{ppm}}$, i[Cys(-Py$_{4F}$)-Gly] = 2 × i_{oF}[Cys(-Py$_{4F}$)-Gly] = 2 × $i_{-92.68\sim-92.91\ \mathrm{ppm}}$, i(GSH) = 2 × [i_{mF}(GSH and Cys)- i_{oF}(Cys)] = 2 × ($i_{-92.37\sim-92.68\ \mathrm{ppm}}$– $i_{-136.17\sim-136.37\ \mathrm{ppm}}$). The high level of γ-GT also hydrolyzed the amide and resulted in the formation of Cys.[6]

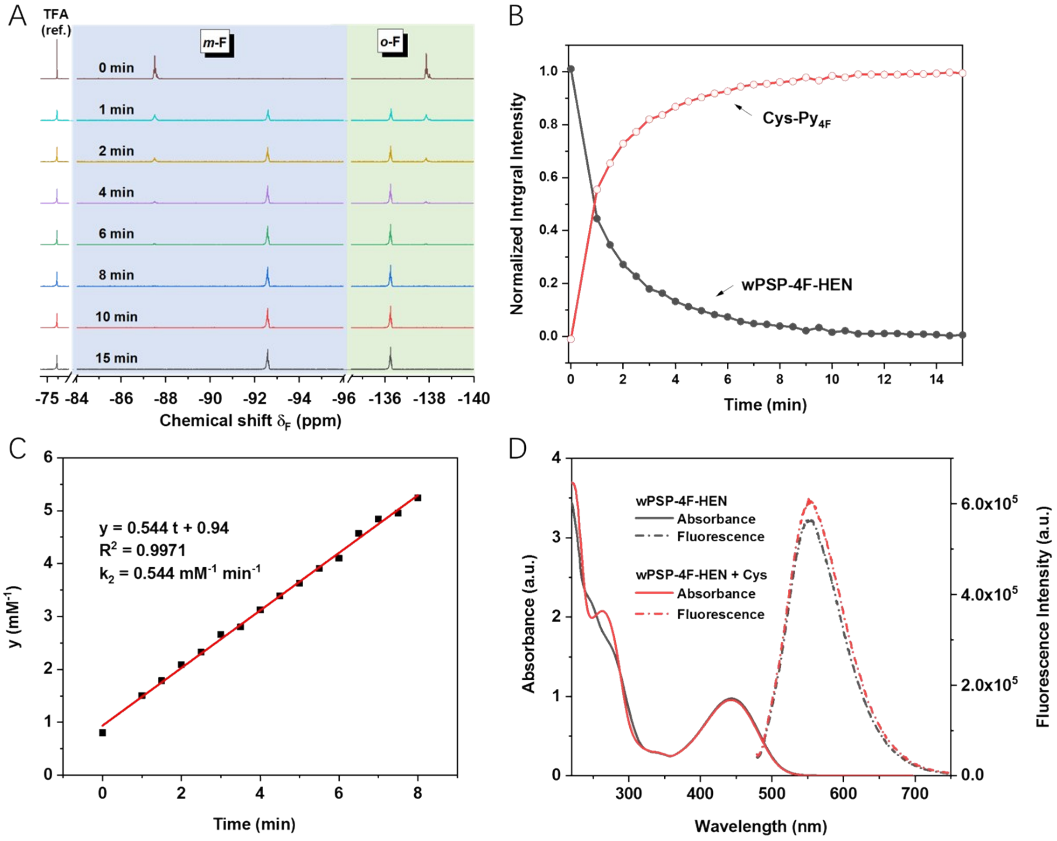

Figure S31 (A) Time-dependent ^{19}F NMR spectra of wPSP-4F-HEN (1.0 mM) upon addition of Cys (1.5 mM) and (B) corresponding integral intensity decay curve in PB (100 mM, pH=7.40, 10 % D$_2$O, 1% DMSO, rt). (C) Y-t

curves. (D) UV−Vis (solid lines) and fluorescence (dash lines, excitation wavelength: 467 nm) spectra of wPSP-4F-HEN (100 μM) before and after addition of Cys (10 mM) in PB (100 mM, pH=7.40, 1% DMSO, rt).

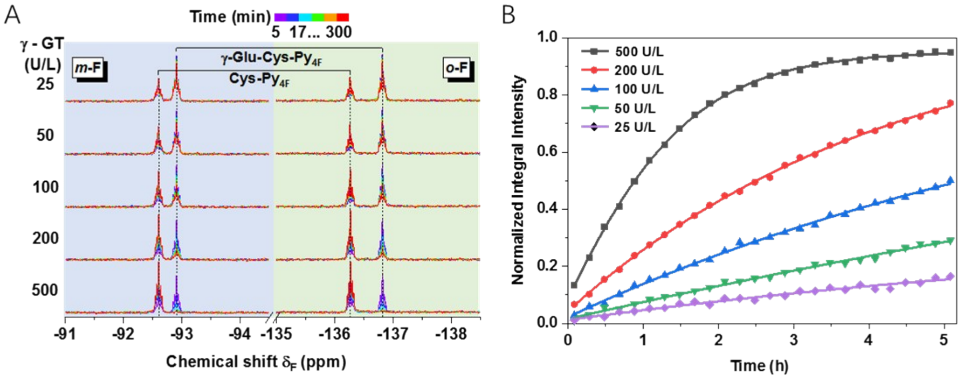

Figure S32 Time-dependent ^{19}F NMR spectra of *γ*-Glu-Cys-Py_{4F} (100 μM) toward *γ*-GT in PB (100 mM, pH=7.40, 10 % D_2O, rt) and (B) corresponding time-course of integral intensities.

4 Synthesis

General Procedure for the Synthesis of Sulfoxide Compounds[7]

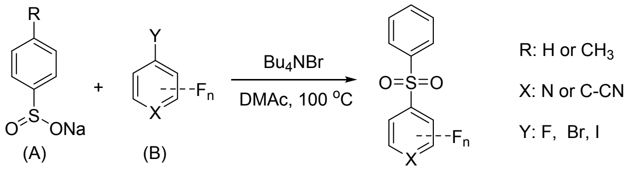

Scheme S1 Synthetic route to sulfoxide compounds.

In a dried Schlenk tube were charged with sodium sulfinate (A), halogenated pyridine (B, 1.0 equiv) and tetrabutylammonium bromide (0.3 equiv) in *N, N*-dimethylacetamide (2 mL per 100 mg of halogenated pyridine) under nitrogen. For **PSP**, **PSP-*o*F**, **PSP-*o*2F** and **PSP-*m*2F**, additional concentrated HCl (1.0 equiv) was added. The mixture was stirred at 100 °C for 24 h and allowed to cool to room temperature. Water was added and the resulting precipitate was collected by filtration. The precipitate was further purified by column chromatography on silica gel to yield the desired product.

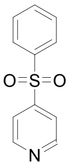

4-(Phenylsulfonyl)pyridine (PSP)

A: sodium benzenesulfinate (1.07 g, 6.49 mmol); B: 4-Bromopyridine hydrochloride (500 mg, 4.33 mmol); **PSP**: white solid (250 mg, 26.3 %). ^{1}H NMR ($CDCl_3$, 600 MHz) δ(ppm): 8.83 (s, 2H), 7.98 (d, 2H, *J* = 7.2 Hz), 7.77 (t, 1H, *J* = 3.6 Hz), 7.64 (t, 2H, *J* = 7.2 Hz), 7.56 (t, 1H, *J* = 6.6 Hz). ^{13}C NMR ($CDCl_3$, 151 MHz) δ(ppm): 151.32, 149.94, 139.81, 134.31, 129.77, 128.28, 120.74. HRMS (ESI, m/z): $[M+H]^+$ calcd for $C_{11}H_{10}NO_2S$: 220.0432; Found, 220.0680.

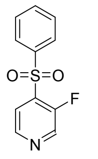

3-Fluoro-4-(phenylsulfonyl)pyridine (PSP-*o*F)

A: sodium benzenesulfinate (177 mg, 1.08 mmol); B: 3-fluoro-4-iodopyridine (200 mg, 4.33 mmol); **PSP-*o*F**: faint yellow solid (70 mg, 35.0 %). ^{1}H NMR ($CDCl_3$, 600 MHz) δ(ppm): 8.66 (d, 1H, J = 4.2 Hz), 8.56 (s, 1H), 8.03 (d, 2H, J = 6.6 Hz), 7.96 (t, 1H, J = 5.4 Hz), 7.69 (t, 1H, J = 6.0 Hz), 7.58 (t, 2H, J = 7.8 Hz). ^{13}C NMR ($CDCl_3$, 151 MHz) δ(ppm): 154.55 (d, J_{CF} = 267.9 Hz), 146.84 (d, J_{CF} = 5.6 Hz), 140.51(d, J_{CF} = 23.7 Hz), 139.40, 136.82(d, J_{CF} = 11.9 Hz), 134.75, 129.61, 128.70, 121.98. ^{19}F NMR ($CDCl_3$, 564 MHz) δ(ppm): -122.96 (d, 1F, J_{HF} = 5.6 Hz). HRMS (ESI, m/z): $[M+H]^+$ calcd for $C_{11}H_9FNO_2S$: 238.0338; Found, 212.0549.

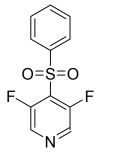

3,5-Difluoro-4-(phenylsulfonyl)pyridine (PSP-*o*2F)

A: sodium benzenesulfinate (165 mg, 1.01 mmol); B: 3,4,5-trifluoropyridine (100 mg, 0.75 mmol); **PSP-*o*2F**: faint yellow solid (98 mg, 38.4 %). ^{1}H NMR ($CDCl_3$, 500 MHz) δ(ppm): 8.47 (s, 2H,), 8.11 (d, 2H, J = 7.5 Hz), 7.72 (t, 1H, J = 7.0 Hz), 7.61 (t, 2H, J = 7.5 Hz). ^{13}C NMR ($CDCl_3$, 126 MHz) δ(ppm): 154.23 (d, J_{CF} = 273.5 Hz), 140.47, 136.65 (d, J_{CF} = 25.1 Hz), 136.56 (d, J_{CF} = 25.1 Hz), 135.14, 129.75, 128.39. ^{19}F NMR ($CDCl_3$, 471 MHz) δ(ppm): -102.39 (s, 2F). HRMS (ESI, m/z): $[M+H]^+$ calcd for $C_{11}H_8F_2NO_2S$: 256.0244; Found, 256.0499.

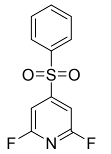

2,6-Difluoro-4-(phenylsulfonyl)pyridine (PSP-*m*2F)

A: sodium benzenesulfinate (306 mg, 1.58 mmol); B: 2,4,6-trifluoropyridine (200 mg, 1.50 mmol); **PSP-*m*2F**: faint yellow solid (106 mg, 27.7 %). ^{1}H NMR ($CDCl_3$, 500 MHz) δ(ppm): 7.99 (d, 2H, J = 8.0 Hz), 7.71 (t, 1H, J = 7.5 Hz), 7.61 (t, 2H, J = 8.0 Hz), 7.30 (s, 2H). ^{13}C NMR ($CDCl_3$, 126 MHz) δ(ppm): 162.39 (dd, J_{CF} = 273.5 Hz and J_{CF} = 14.4 Hz), 159.41 (t, J_{CF} = 6.3 Hz), 138.79, 135.24, 130.31, 128.77, 105.22 (dd, J_{CF} = 30.1 Hz and J_{CF} = 13.6 Hz), ^{19}F NMR ($CDCl_3$, 471 MHz) δ(ppm): -62.21(s, 2F). HRMS (ESI, m/z): $[M+H]^+$ calcd for $C_{11}H_8F_2NO_2S$: 256.0244; Found, 256.0381.

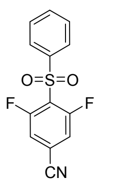

3,5-Difluoro-4-(phenylsulfonyl)benzonitrile (PSBN-*o*2F)

A: sodium benzenesulfinate (389 mg, 2.37 mmol); B: 3,4,5-trifluorobenzonitrile (400 mg, 2.26 mmol); **PSBN-*o*2F**: faint yellow solid (228 mg, 36.2 %). ^{1}H NMR ($CDCl_3$, 500 MHz) δ(ppm): 8.09 (d, 2H, J = 7.5 Hz), 7.71 (t, 1H, J = 7.5 Hz), 7.60 (t, 2H, J = 7.5 Hz), 7.30 (d, 2H, J = 7.5 Hz). ^{13}C NMR ($CDCl_3$, 126 MHz) δ(ppm): 160.10 (dd, J_{CF} = 265.1 Hz and J_{CF} = 4.3 Hz), 141.00, 135.21, 129.92, 128.45, 119.00 (t, J_{CF} = 12.2 Hz),117.62 (d J_{CF} = 27.8 Hz), 117.59 (d J_{CF} = 27.8 Hz), 115.41. ^{19}F NMR ($CDCl_3$, 471 MHz) δ(ppm): -101.53 (d, 2F, J_{HF} = 8.0 Hz). HRMS (ESI, m/z): $[M+H]^+$ calcd for $C_{13}H_8F_2NO_2$: 280.0244; Found, 280.0552.

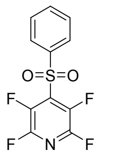

2,3,5,6-Tetrafluoro-4-(phenylsulfonyl)pyridine (PSP-4F)

A: sodium benzenesulfinate (1.94 g, 11.83 mmol); B: perfluoropyridine (2.00 g, 11.83 mmol); **PSP-4F**: white solid (2.70 g, 78.37 %). ^{1}H NMR ($CDCl_3$, 500 MH-z) δ(ppm): 8.13 (d, 2H, J = 8.0 Hz), 7.78 (t, 1H, J = 7.5 Hz), 7.65 (t, 2H, J = 7.5 Hz). ^{13}C NMR ($CDCl_3$, 126 MHz) δ(ppm): 145.24 (dm, J_{CF} = 250.6 Hz), 139.33, 138.95 (dm, J_{CF} = 272.9 Hz), 135.84, 133.26 (t, J_{CF} = 13.0 Hz), 130.07, 128.64. ^{19}F NMR ($CDCl_3$, 471 MHz) δ(ppm): -85.76 (m, 2F), -137.01 (m, 2F). HRMS (ESI, m/z): $[M+H]^+$ calcd for $C_{11}H_6F_4NO_2S$: 292.0055; Found, 256.0220.

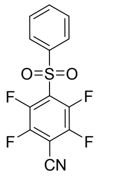

2,3,5,6-Tetrafluoro-4-(phenylsulfonyl)benzonitrile (PSBN-4F)

A: sodium benzenesulfinate (170 mg, 1.04 mmol); B: 2,3,4,5,6-pentafluorobenzonitrile (200 mg, 1.04 mmol); **PSBN-4F**: faint yellow solid (140 mg, 42.9 %). ^{1}H NMR ($CDCl_3$, 500 MHz) δ(ppm): 8.10 (d, 2H, J = 7.5 Hz), 7.76 (t, 1H, J = 7.5 Hz), 7.64 (t, 2H, J = 7.5 Hz). ^{13}C NMR ($CDCl_3$, 126 MHz) δ(ppm): 147.88 (dm, J_{CF} = 265.7 Hz), 144.42 (dm, J_{CF} = 264.6 Hz),140.04, 135.94, 130.27, 128.64, 127.36 (t, J_{CF} =14.2 Hz), 106.54 (t, J_{CF} = 3.3 Hz), 99.18 (t, J_{CF} = 17.1 Hz). ^{19}F NMR ($CDCl_3$, 471 MHz) δ(ppm): -128.48 (m, 2F), -132.89 (m, 2F). HRMS (ESI, m/z): $[M+H]^+$ calcd for $C_{13}H_6F_4NO_2S$:316.0055; Found, 316.0232.

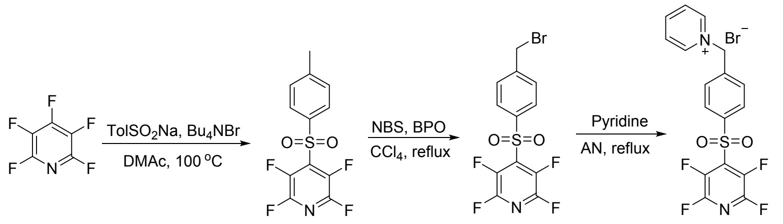

C1 C2 wPSP-4F

Scheme S2 Synthetic route to wPSP-4F.

2,3,5,6-Tetrafluoro-4-tosylpyridine (C1)

Following general procedure for the synthesis of sulfoxide compounds. A: sodium *p*-toluenesulfinate (1.11 g, 6.21 mmol); B: perfluoropyridine (1.00 g, 5.92 mmol); **C1**: white solid (1.55 g, 85.8 %). ^{1}H NMR ($CDCl_3$, 500 MHz) δ(ppm): 7.99 (d, 2H, J = 7.5 Hz), 7.43 (d, 2H, J = 8.5 Hz). ^{13}C NMR ($CDCl_3$, 126 MHz) δ(ppm): 147.50, 144.41 (dm, J_{CF} = 250.5 Hz), 138.75 (dm, J_{CF} = 273.7 Hz), 136.839, 133.60 (t, J_{CF} = 13.0 Hz), 130.68, 128.69. ^{19}F NMR ($CDCl_3$, 471 MHz) δ(ppm): -86.01 (m, 2F), -137.18 (m, 2F). HRMS (ESI, m/z): $[M+H]^+$ calcd for $C_{12}H_8F_4NO_2S$: 306.0212; Found, 306.0475.

4-((4-(bromomethyl)phenyl)sulfonyl)-2,3,5,6-tetrafluoropyridine (C2)

In a dried Schlenk tube were charged with **C1** (500 mg, 1.64 mmol), *N*-bromosuccinimide (306 mg, 1.72) and

benzoyl peroxide (20 mg, 0.082 mmol) in CCl_4 (15 mL) under nitrogen. The reaction mixture was refluxed overnight and then poured into water. The mixture was extracted with DCM for three times. The organic layer was collected and dried over anhydrous sodium sulfate. After removal of the solvent under reduced pressure, the crude product was purified by column chromatography on silica gel to yield a white solid as the desired product (240 mg, 38.1 %). ^{1}H NMR ($CDCl_3$, 500 MHz) δ(ppm): 8.09 (d, 2H, J = 8.0 Hz), 7.66 (d, 2H, J = 8.0 Hz), 4.51 (s, 2H). ^{13}C NMR ($CDCl_3$, 126 MHz) δ(ppm): 146.15, 144.30 (dm, J_{CF} = 241.9 Hz),138.96, 138.81 (dm, J_{CF} = 268.4 Hz), 133.01 (t, J_{CF} = 12.6 Hz), 130.59, 129.23, 30.87. ^{19}F NMR ($CDCl_3$, 471 MHz) δ(ppm): 85.46 (m, 2F), 136.87 (m, 2F). HRMS (ESI, m/z): $[M+H]^+$ calcd for $C_{12}H_7BrF_4NO_2S$: 383.9317; Found, 383.9592.

1-(4-((Perfluoropyridin-4-yl)sulfonyl)benzyl)pyridin-1-ium bromide (wPSP-4F)

In a dried Schlenk tube were charged with **C2** (105 mg, 0.27 mmol) and pyridine (26 mg, 0.33 mmol) in acetonitrile (3 mL) under nitrogen. The reaction mixture was refluxed overnight and allowed to cool to room temperature. The resulting precipitate was collected by filtration and washed fully with DCM to yield a yellow solid as the desired product (100 mg, 78.8 %). ^{1}H NMR (MeOD-d_4, 600 MHz) δ(ppm): 9.12 (d, 2H, J = 6.0 Hz), 8.67 (t, 1H, J = 7.8 Hz), 8.20 (m, 4H), 7.81 (d, 2H, J = 8.4 Hz), 6.03 (s, 2H). ^{13}C NMR (MeOD-d_4, 126 MHz) δ(ppm): 147.83, 146.52, 145.52 (dm, J_{CF} = 248.1 Hz), 142.47, 141.91, 140.38 (dm, J_{CF} = 271.2 Hz), 133.31 (t, J_{CF} = 12.6 Hz), 131.40, 130.65, 130.01, 64.49. ^{19}F NMR (MeOD-d_4, 564 MHz) δ(ppm):89.62 (m, 2F), 139.61 (m, 2F). HRMS (ESI, m/z): $[M-Br]^+$ calcd for $C_{17}H_{11}F_4N_2O_2S$: 383.0472; Found, 383.0619.

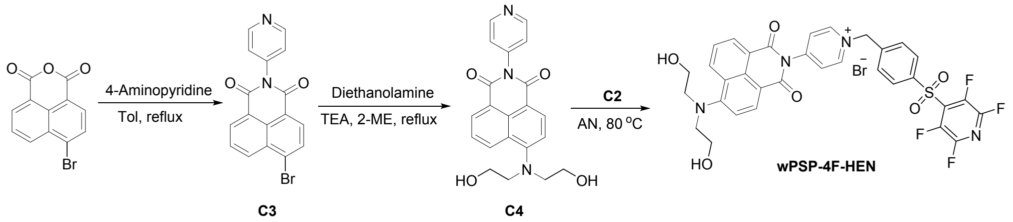

Scheme S3 Synthetic route to wPSP-4F-HEN

6-Bromo-2-(pyridin-4-yl)-1H-benzo[de]isoquinoline-1,3(2H)-dione (C3)

In a round-bottomed flask were added 6-bromo-1H,3H-benzo[de]isochromene-1,3-dione (1.00 g, 0.36 mmol) and (0.51 g, 0.54 mmol) in anhydrous Tol (25 mL). The reaction mixture was refluxed overnight. After cooling to room temperature, the mixture was diluted with DCM and the insoluble substances were removed through filtration. The solvent was then removed under reduced pressure to get the crude product. The product was further recrystallized from EtOH to yield a faint pink solid as the desired product (450 mg, 35.3 %). ^{1}H NMR ($CDCl_3$, 500 MHz)

δ(ppm): 8.84 (d, 2H, J = 5.5 Hz), 8.72 (d, 1H, J = 7.0 Hz), 8.68 (d, 1H, J = 8.5 Hz), 8.47 (d, 1H, J = 7.5 Hz), 8.12 (d, 1H, J = 7.5 Hz), 7.91 (t, 1H, J = 8.0 Hz), 7.32 (d, 2H, J = 5.5 Hz). ^{13}C NMR ($CDCl_3$, 126 MHz) δ(ppm): 163.20, 163.17, 151.28, 143.15, 134.27, 132.87, 131.98, 131.51, 131.41, 131.04, 129.48, 128.45, 124.12, 122.90, 121.99. HRMS (ESI, m/z): $[M+H]^+$ calcd for $C_{17}H_{10}BrN_2O_2$: 352.9926; Found, 353.0316.

6-(Bis(2-hydroxyethyl)amino)-2-(pyridin-4-yl)-1H-benzo[de]isoquinoline-1,3(2H)-dione (C4)

In a round-bottomed flask were added **C3** (400 mg, 1.13 mmol), diethanolamine (595 mg, 5.66 mmol) and triethylamine (1 mL) in 2-Methoxyethanol (5 mL). The reaction mixture was refluxed overnight. After solvent removal under reduced pressure, the crude product was purified by column chromatography to yield an orange red solid as the desired product (108 mg, 25.3 %). ^{1}H NMR (MeOD-d_4, 500 MHz) δ(ppm): 8.89 (d, 1H, J = 8.0 Hz), 8.74 (d, 2H, J = 5.5 Hz), 8.58 (d, 1H, J = 7.0 Hz), 8.50 (d, 1H, J = 8.5 Hz), 7.78 (t, 1H, J = 7.5 Hz), 7.52 (m, 3H), 3.81 (t, 4H, J = 5.5 Hz), 3.70 (t, 4H, J = 5.5 Hz). ^{13}C NMR ($CDCl_3$, 126 MHz) δ(ppm): 166.16, 165.55, 164.99, 157.95, 151.28, 146.54, 133.70, 132.64, 132.14, 128.41, 126.43, 126.40, 123.91, 118.27, 116.15, 60.32, 56.89. HRMS (ESI, m/z): $[M+H]^+$ calcd for $C_{21}H_{20}N_3O_4$: 378.1454; Found, 378.1821.

4-(6-(Bis(2-hydroxyethyl)amino)-1,3-dioxo-1H-benzo[de]isoquinolin-2(3H)-yl)-1-(4-((perfluoropyridin-4-yl)sulfonyl)benzyl)pyridin-1-ium bromide (wPSP-4F-HEN)

In a dried Schlenk tube were charged with **C2** (81 mg, 0.21 mmol) and **C4** (80 mg, 0.21 mmol) in acetonitrile (3 mL) under nitrogen. The reaction mixture was stirred at 80 ^{o}C overnight and allowed to cool to room temperature. The resulting precipitate was collected by filtration and washed fully with DCM to yield an orange red solid as the desired product (72 mg, 45.0 %). ^{1}H NMR (MeOD-d_4, 500 MHz) δ(ppm): 9.27 (d, 2H, J = 7.0 Hz), 8.92 (d, 1H, J = 8.5 Hz), 8.62 (d, 1H, J = 7.5 Hz), 8.52 (d, 1H, J = 8.5 Hz), 8.35 (d, 2H, J = 6.5 Hz), 8.26 (d, 2H, J = 8.0 Hz), 7.89 (d, 2H, J = 8.0 Hz), 7.80 (t, 1H, J = 8.0 Hz), 7.52 (d, 1H, J = 8.0 Hz), 6.09 (s, 2H), 3.81 (t, 4H, J = 5.0 Hz), 3.72 (t, 4H, J = 5.5 Hz). ^{13}C NMR ($CDCl_3$, 126 MHz) δ(ppm): 164.94, 164.18, 158.66, 154.35, 147.29, 145.54 (dm, J_{CF} = 249.5 Hz), 142.29, 142.15, 140.56 (dm, J_{CF} = 270.5 Hz), 134.40, 134.27, 133.36 (dm, J_{CF} = 13.2 Hz), 133.11, 132.34, 131.55, 131.12, 130.77, 128.13, 126.35, 123.32, 118.03, 115.00, 64.42, 60.26, 56.81. ^{19}F NMR (MeOD-d_4, 471 MHz) δ(ppm):89.62 (m, 2F), 139.55 (m, 2F). HRMS (ESI, m/z): $[M-Br]^+$ calcd for $C_{33}H_{25}F_4N_4O_6$: 681.1425; Found, 681.1492.

5 Reference

1 J. Axthelm, H. Görls, U. S. Schubert and A. Schiller, *J. Am. Chem. Soc.*, 2015, **137**, 15402-15405.

2 Y. Zheng, H. Liu, G. Ma, P. Yang, L. Zhang, Y. Gu, Q. Zhu, T. Shao, P. Zhang, Y. Zhu and W. Cai, *J. Pharm. Biomed. Anal.*, 2011, **54**, 1187-1191.

3 H. Zhang, R. Liu, J. Liu, L. Li, P. Wang, S. Q. Yao, Z. Xu and H. Sun, *Chem. Sci.*, 2016, **7**, 256-260.

4 S. Kalhor-Monfared, M. R. Jafari, J. T. Patterson, P. I. Kitov, J. J. Dwyer, J. M. Nuss and R. Derda, *Chem. Sci.*, 2016, **7**, 3785-3790.

5 G. Lautrette, F. Touti, H. G. Lee, P. Dai and B. L. Pentelute, *J. Am. Chem. Soc.*, 2016, **138**, 8340-8343.

6 J. Liu, S. Zhang, B. Zhao, C. Shen, X. Zhang and G. Yang, *Biosens. Bioelectron.*, 2019, **142**, 111497.

7 K. M. Maloney, J. T. Kuethe and K. Linn, *Org. Lett.*, 2011, **13**, 102-105.
